# HiCcompare: an R-package for joint normalization and comparison of HI-C datasets

**DOI:** 10.1186/s12859-018-2288-x

**Published:** 2018-07-31

**Authors:** John C. Stansfield, Kellen G. Cresswell, Vladimir I. Vladimirov, Mikhail G. Dozmorov

**Affiliations:** 1 Department of Biostatistics, Virginia Commonwealth University, Richmond, VA 23298 USA; 2 Department of Psychiatry, Virginia Institute for Psychiatric and Behavioral Genetics, Richmond, VA 23219 USA

**Keywords:** Hi-C, Chromosome conformation capture, Normalization, Comparison, Differential analysis, HiCcompare

## Abstract

**Background:**

Changes in spatial chromatin interactions are now emerging as a unifying mechanism orchestrating the regulation of gene expression. Hi-C sequencing technology allows insight into chromatin interactions on a genome-wide scale. However, Hi-C data contains many DNA sequence- and technology-driven biases. These biases prevent effective comparison of chromatin interactions aimed at identifying genomic regions differentially interacting between, e.g., disease-normal states or different cell types. Several methods have been developed for normalizing individual Hi-C datasets. However, they fail to account for biases *between two or more Hi-C datasets*, hindering comparative analysis of chromatin interactions.

**Results:**

We developed a simple and effective method, HiCcompare, for the joint normalization and differential analysis of multiple Hi-C datasets. The method introduces a distance-centric analysis and visualization of the differences between two Hi-C datasets on a single plot that allows for a data-driven normalization of biases using locally weighted linear regression (loess). HiCcompare outperforms methods for normalizing individual Hi-C datasets and methods for differential analysis (diffHiC, FIND) in detecting a priori known chromatin interaction differences while preserving the detection of genomic structures, such as A/B compartments.

**Conclusions:**

HiCcompare is able to remove between-dataset bias present in Hi-C matrices. It also provides a user-friendly tool to allow the scientific community to perform direct comparisons between the growing number of pre-processed Hi-C datasets available at online repositories. HiCcompare is freely available as a Bioconductor R package https://bioconductor.org/packages/HiCcompare/.

**Electronic supplementary material:**

The online version of this article (10.1186/s12859-018-2288-x) contains supplementary material, which is available to authorized users.

## Background

The 3D chromatin structure of the genome is emerging as a unifying regulatory framework orchestrating gene expression by bringing transcription factors, enhancers and co-activators in spatial proximity to the promoters of genes [1–4]. Changes in chromatin interactions shape cell type-specific gene expression [5–8], as well as misregulation of oncogenes and tumor suppressors in cancer [9–11] and other diseases [3]. Identifying changes in chromatin interactions is the next logical step in understanding genomic regulation.

Evolution of Chromatin Conformation Capture (3C) technologies into Hi-C sequencing now allows the detection of “all vs. all” long-distance chromatin interactions across the whole genome [6, 12]. Soon after public Hi-C datasets became available, it was clear that technology- and DNA sequence-driven biases substantially affect chromatin interactions [13]. The technology-specific biases include the cutting length of a restriction enzyme (HindIII, MboI, or NcoI), cross-linking conditions, circularization length, etc. The DNA sequence-driven biases include GC content, mappability, nucleotide composition. Discovery of these biases led to the development of methods for normalizing individual datasets [6, 13–16]. Although normalization of individual datasets improves reproducibility within replicates of Hi-C data [13, 15], these methods do not consider biases between multiple Hi-C datasets.

Accounting for the between-dataset biases is critical for the correct identification of chromatin interaction changes between, e.g., disease-normal states, or cell types. If between dataset biases (due to technology, batch effects, processing, etc.) are left unchecked, biases can be mistaken for biologically relevant differential interactions. While DNA sequence-driven biases affect two datasets similarly (e.g., GC content of genomic regions tested for interaction differences is the same), technology-driven biases are poorly characterized and affect chromatin interactions unpredictably between Hi-C libraries. Importantly, another source of chromatin interaction differences stems from large-scale genomic rearrangements, such as copy number variations [17], a frequent event in cancer genomes [18]. Accounting for such biases is needed for the accurate detection of differential chromatin interactions between Hi-C datasets.

We developed an R package, HiCcompare, for the joint normalization and comparative analysis of processed Hi-C datasets. Our method is based on the observation that chromatin interactions are highly stable [7, 19–21], suggesting that the majority of them can serve as a reference to build a rescaling model. We present the novel concept of the MD plot (*M*inus, or difference vs. *D*istance plot), a modification of the MA plot [22]. The MD plot allows for visualizing the differences between interacting chromatin regions in two Hi-C datasets while explicitly accounting for the linear distance between interacting regions. The MD plot concept naturally allows for fitting the local regression model, a procedure termed loess, and jointly normalizing the two datasets by balancing biases between them. The distance-centric view of chromatin interaction differences allows for detecting statistically significant differential chromatin interactions between two Hi-C datasets. We show improved performance of differential chromatin interaction detection when using the jointly vs. individually normalized Hi-C datasets. Our method is broadly applicable to a range of biological problems, such as identifying differential chromatin interactions between tumor and normal cells, immune cell types, and normal tissues/cell types.

## Implementation

HiCcompare is implemented as a Bioconductor R package. All functions are written in R and vectorized where possible for the greatest computational speed. The biggest advantage of loess - the ability to model any biases in the data without explicitly specifying them - comes at the cost of increased computation. The Bioconductor BiocParallel package was used to implement parallel processing for the normalization and comparison steps on a chromosome-specific basis. If enough cores are available, such as on a computing cluster, each chromosome’s normalization and comparison steps can be sent to their own processor for analysis, improving the total run time (Additional file 1: Figure 3.1).

**Fig. 1:**
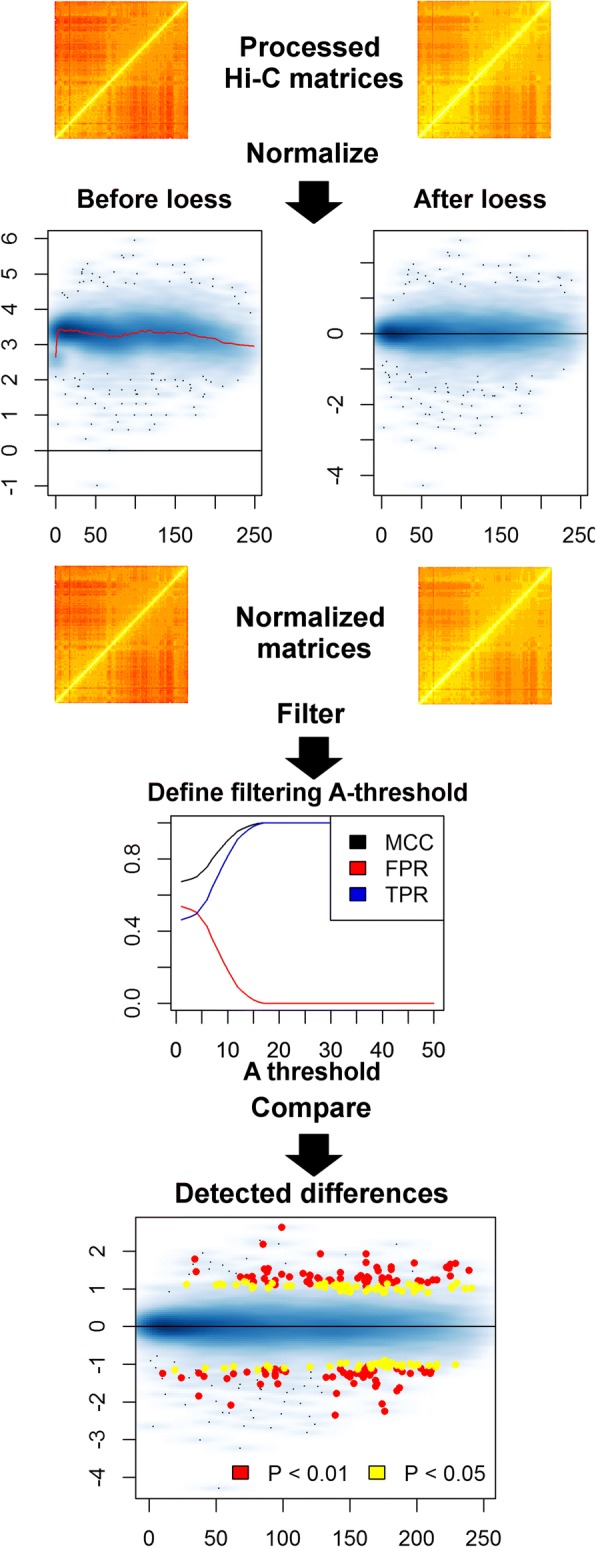
HiCcompare flow chart. Processed Hi-C libraries in the form of sparse upper triangular matrices are the starting data type for HiCcompare. Data is then plotted on the MD plot, and a loess model is fit to remove bias between the libraries. Next, the filtering threshold needs to be determined. Finally, the libraries can be compared for differences and plotted again on the MD plot

Additionally, the package includes vignettes with test data and documentation for all functions, as well as code to generate the results referenced in this manuscript. The general workflow of a HiCcompare analysis is diagrammed in the flow chart (Fig. 1). HiCcompare can be run interactively on a laptop to analyze a single pair of chromatin interaction matrices or utilized in a script for analyzing the entire genome in parallel on a cluster. HiCcompare is released under the MIT open-source software license.

## Results and discussion

### Hi-C data representation and properties

**Fig. 2:**
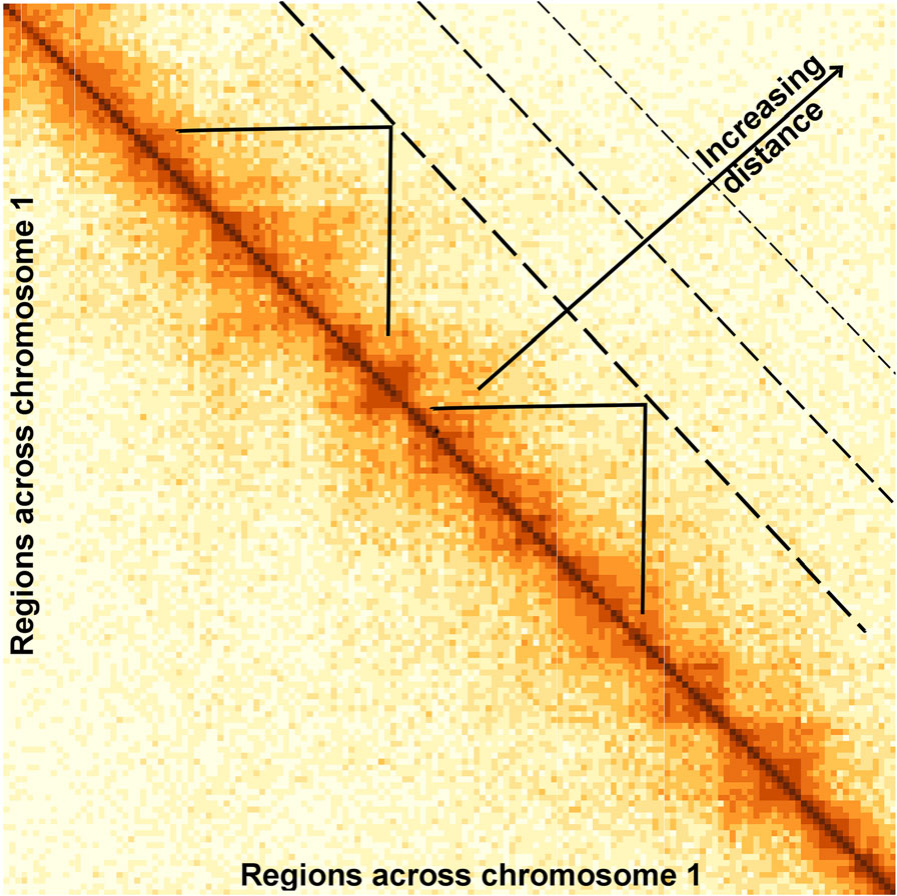
Distance-centric (off-diagonal) view of chromatin interaction matrices. Each off-diagonal vector of interaction frequencies represents interactions at a given distance between pairs of regions. Triangles mark pairs of genomic regions interacting at the same distance. Data for chromosome 1, K562 cell line, 50 KB resolution, spanning 0–7.5 Mb is shown

HiCcompare focuses on the joint analysis of multiple Hi-C datasets represented by chromatin interaction matrices, where rows and columns represent genomic regions (bins), and cells contain interaction counts (frequencies). A chromosome-specific Hi-C matrix is a square matrix of size *N* × *N*, where *N* is the number of genomic regions (bins) of size *X* on a chromosome. The size *X* of the genomic regions defines the resolution of the Hi-C data. Each cell in the matrix contains an interaction frequency *IF*_*i*, *j*_, where *i* and *j* are the indices of the interacting regions. The values on the diagonal trace represent interaction frequencies (IFs) of self-interacting regions. Each off-diagonal trace of values represents interaction frequencies for a pair of regions at a given unit-length distance. The unit-length distance is expressed in terms of resolution of the data (the size of genomic regions, typically measured in millions (thousands) of base pairs, MB (KB)). The concept of considering interaction frequencies at each off-diagonal trace is central for the joint normalization and differential chromatin interaction detection (Fig. 2).

The interaction frequency drops as the distance between interacting regions increases. Numerous attempts have been made to parametrically model the inverse relationship between chromatin interaction frequency and the distance between interacting regions. However, Hi-C data are affected by technology- and DNA sequence-driven biases [13–15], unpredictably altering chromatin interaction frequencies. Consequently, parametric approaches fail to model interaction frequencies across the full range of distances [12], confirmed by our observations (Additional file 1: Figure 2.1). For this study, data in the sparse upper triangular format from the GM12878, K562, and RWPE1 cell lines were used (Supplemental Methods, Additional file 1).

It is also important to note that HiCcompare is designed to analyze pre-processed Hi-C data, unlike many other tools which require the user to deal with the raw sequencing data. There are a growing number of Hi-C libraries, already processed into matrix format, available for download on many public repositories such as GEO. HiCcompare is specifically designed to make it easy for the user to perform their own analyses on these pre-processed Hi-C matrices.

### Visualization of the differences between two Hi-C datasets

The first step of the HiCcompare procedure is to convert the data into what we refer to as an MD plot. The MD plot is similar to the MA plot (Bland-Altman plot) commonly used to visualize gene expression differences [22]. *M* is defined as the log difference between the two data sets *M* = *log*_2_(*IF*_2_/*IF*_1_), where *IF*_1_ and *IF*_2_ are interaction frequencies of the first and the second Hi-C datasets, respectively. *D* is defined as the distance between two interacting regions, expressed in unit-length of the *X* resolution of the Hi-C data. In terms of chromatin interaction matrices, *D* corresponds to the off-diagonal traces of interaction frequencies (Fig. 2). Because chromatin interaction matrices are sparse, i.e., contain an excess of zero interaction frequencies, and it cannot be determined if a zero IF represents missing data or a true absence of interaction, by default only the non-zero pairwise interaction are used for the construction of the MD plot. However, if the user wishes to include partial zero interactions, i.e. with a zero value in one of the matrices and a non-zero IF in the other the option is available.

### Elimination of biases in jointly, but not individually, normalized Hi-C data

Discovery of biases in Hi-C data led to the development of numerous methods for normalizing *individual* datasets [6, 14–16]. Although normalization of individual datasets improves reproducibility of replicated Hi-C data [13, 15], these methods focus on correcting biological and internal biases and do not explicitly account for biases between *multiple* Hi-C datasets. When the goal is to compare two Hi-C libraries it can be assumed that many of these internal and biological biases affect both libraries similarly and thus their correction is less important. It is the between-dataset biases that are particularly problematic when comparing Hi-C datasets between biological conditions (Section 4, Additional file 1). To detect chromatin interaction differences due to biology, not biases, it is critical to use a normalization method that removes the between-dataset biases.

**Fig. 3:**
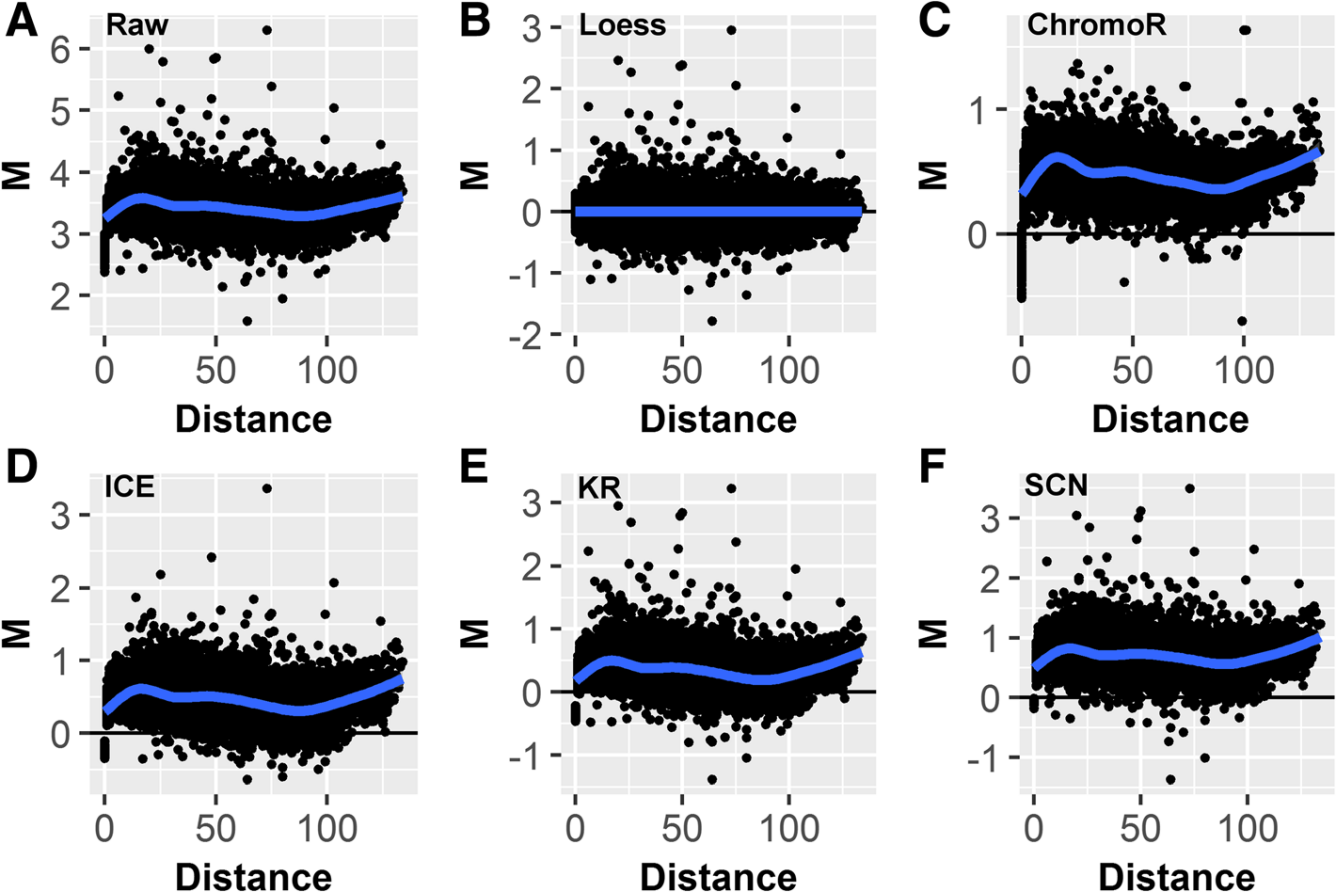
MD plot data visualization and the effects of different normalization techniques. MD plots of the differences M between two replicated Hi-C datasets (GM12878 cell line, chromosome 11, 1 MB resolution, DpnII and MboI restriction enzymes) plotted vs. distance D between interacting regions. **a** Before normalization, **b** after loess joint normalization, **c** ChromoR, **d** Iterative Correction and Eigenvector decomposition (ICE), **e** Knight-Ruiz (KR), **f** Sequential Component Normalization (SCN). The general shift of the data above M = 0 is due to one of the Hi-C libraries having more total reads. The trends emphasized by the loess curve imposed on the data are due to distance dependent between-dataset biases which only HiCcompare’s joint normalization procedure can successfully remove

To assess the between-dataset biases, we visualize two Hi-C datasets on a single MD plot. Visualizing replicates of Hi-C data (Gm12878 cell line) showed the presence of biases in the individually normalized datasets (Fig. 3 and Section 4, Additional file 1), suggesting that the performance of individual normalization methods may be sub-optimal when comparing multiple Hi-C datasets.

To account for between-dataset biases, we developed a non-parametric joint normalization method that makes no assumptions about the theoretical distribution of the chromatin interaction frequencies. It utilizes the well-known loess (locally weighted polynomial regression) smoothing algorithm - a regression-based method for fitting simple models to segments of data [23]. The main advantage of loess is that it accounts for any local irregularities *between* the datasets that cannot be modeled by parametric methods. Thus, loess is particularly appealing when normalizing two Hi-C datasets, as the internal biases in Hi-C data are poorly understood (Fig. 3).

The HiCcompare joint normalization procedure proceeds by first plotting the data on the MD plot, then loess regression [23] is performed with *D* as the predictor for *M*. The fitted values are then used to normalize the original IFs:

$$ \left\{\begin{array}{l} lo{g}_2\left({\hat{IF}}_{1D}\right)= lo{g}_2\left(I{F}_{1D}\right)+f(D)/2\\ {} lo{g}_2\left({\hat{IF}}_{2D}\right)= lo{g}_2\left(I{F}_{2D}\right)-f(D)/2\end{array}\right. $$

where *f*(*D*) is the predicted value from the loess regression at a distance *D*. The $lo{g}_2\left(\hat{IF}\right)$ values are then anti-logged to obtain the normalized IFs. Note that for both Hi-C datasets the average interaction frequency remains unchanged, as *IF*_1_ is increased by the factor of *f*(*D*)/2 while *IF*_2_ is decreased by the same amount. Any normalized IFs with values less than one are not considered in further analyses. The joint normalization was tested against five methods for normalizing individual Hi-C matrices, ChromoR [24], ICE [15], KR [16], SCN [14], MA [25] (Supplemental Methods, Additional file 1).

Existing Hi-C data at high resolutions (e.g., 10 kb) still suffer from a limited dynamic range of chromatin interaction frequencies, with the majority of them being small or zero, especially at large distances between interacting regions. This sparsity places limits on loess joint normalization, as it builds a rescaling model from many non-zero pairwise comparisons. A way to alleviate this limitation is to consider interactions only within a range of short interaction distances, where genomic regions interact more frequently, and the proportion of zero interaction frequencies is the lowest. Our evaluation of loess joint normalization showed it performs best at resolutions between 1 MB and 50 KB (Section 4 & Section 7, Additional file 1). The issue of sparsity limiting the usefulness of loess normalization will be alleviated as sequencing techniques continue to improve and Hi-C datasets with deeper sequencing become available.

### Excluding potentially problematic regions from the joint normalization

Some between-dataset biases may occur due to large-scale genomic rearrangements and copy number variants (CNVs), a frequent case in tumor-normal comparisons [18]. Similar to removing other biases, the joint loess normalization removes CNV-driven biases by design, allowing for the detection of chromatin interaction differences within CNV regions. However, CNVs introduce large changes in chromatin interactions [17], which may be of interest to consider separately. Therefore, unless cells/tissues with normal karyotypes are compared, we provide optional functionality for the detection and removal of genomic regions containing CNVs from the joint normalization. The QDNAseq [26] R package is used to detect and exclude CNVs from the HiCcompare analysis. Alternatively, CNV regions can be detected separately and provided to HiCcompare as a BED file. Additionally, the HiCcompare package includes the ENCODE blacklisted regions for hg19 and hg38 genome assemblies, which can be excluded from further analysis.

### Detecting differential chromatin interactions

After joint normalization, the chromatin interaction matrices are ready to be compared for differences. Again, the MD plot is used to represent the differences *M* between two normalized datasets at a distance *D*. The jointly normalized *M* values are centered around 0 and are approximately normally distributed across all distances (Supplemental Methods, Additional file 1). *M* values can be converted to Z-scores using the standard approach:

$$ {Z}_i=\frac{M_i-\overline{M}}{\sigma_M} $$

where $\overline{M}$ is the mean value of all *M*’s on the chromosome and *σ*_*M*_ is the standard deviation of all *M* values on the chromosome and *i* is the *i*th interacting pair on the chromosome.

During Z-score conversion, the average expression of each interacting pair is considered. Due to the nature of *M*, a difference represented by an interacting pair with IFs 1 and 10 is equivalent to an interacting pair of IFs 10 and 100 with both differences producing an *M* value of 3.32. However, the average expression of these two differences is 5.5 and 55, respectively. Differences with higher average expression are supported by the larger number of sequencing reads and are therefore more trustworthy than the low average expression differences. Thus, we filter out differences with low average expression by setting the Z-scores to 0 when average expression (*A*) is less than a user set value of *A* (Supplemental Methods, Additional file 1). Filtering takes place such that the $\overline{M}$ and *σ*_*M*_ are calculated using only the *M* values remaining after filtering. The Z-scores can then be converted to *p*-values using the standard normal distribution.

Analyzing Hi-C data for differences necessarily involves testing of multiple hypotheses. Multiple testing correction (False Discovery Rate (FDR)) is applied on a per-distance basis by default, with an option to apply it on a chromosomal basis. If a method other than FDR is desired, all other standard multiple testing corrections are available for the user to choose from.

As there is no “gold standard” for differential chromatin interactions, we created such a priori known differences by introducing controlled changes to replicate Hi-C datasets [27]. To introduce these a priori known differences, we start with two replicates of Hi-C data from the same cell type. It is assumed that any differences in these replicates are due to noise or technical biases. Next, we randomly sample a specified number of entries in the contact matrix. These sampled entries are where the changes will be introduced. The IFs for each of these entries in the two matrices are set to their average value between the replicates, and then one of them is multiplied by a specified fold change. This introduces a true difference at an exact fold change between the two replicates. The benefit of using joint normalization vs. individually normalized datasets was quantified by the improvement in power of detecting the pre-defined chromatin interaction differences. Standard classifier performance measures (Section “Availability and requirements”, Additional file 1), summarized in the Matthews Correlation Coefficient (MCC) metric, were assessed. HiCcompare is able to detect most of the added differences with a relatively low number of false positives across the range of fold changes (Table 1, Section “Availability and requirements”, Additional file 1).

**Table 1:** Evaluation of the effect of normalization on differential chromatin interaction detection

Fold change	HiCcompare	MA	ICE	SCN	KR	ChromoR
2	0.847	0.823	0.835	0.768	0.748	0.149
3	0.973	0.934	0.802	0.721	0.764	0.380
4	0.995	0.98	0.953	0.881	0.868	0.532

Matthews Correlation Coefficient of detecting 200 controlled differences in jointly (HiCcompare) vs. individually normalized Gm12878 datasets, chromosome 1, 1 MB resolution. Matrices were normalized with methods corresponding to column labels; differences were detected using HiCcompare

### Differential regions overlap with CTCF sites

We hypothesized that regions, detected as differentially interacting, most likely represent biologically relevant boundaries of topologically associated domains changing between two conditions. As such, we investigated whether differentially interacting regions are enriched in CTCF binding sites, an insulator protein known to bind at TAD boundaries [28]. To test that, we compared Hi-C data from GM12878 and K562 cell lines at 100 MB resolution using HiCcompare. A total of 2365 interactions were identified as interacting differentially (FDR < 0.05) which represented 2783 distinct 100 KB genomic regions. We found that a total of 130,675 CTCF binding sites overlapped with these regions. The amount of overlaps observed was significant (permutation *p*-value = 0.002), confirming our hypothesis that the differentially interacting regions detected by HiCcompare play an import biological role in chromatin structural organization.

### Example HiCcompare analysis using mouse neuronal differentiation

As an example case for the usage of HiCcompare, we performed an analysis to compare the 3D structure of the chromatin between mouse embryonic stem cells (ESC), neural progenitor cells (NPC), and neurons. The data was obtained from a study by Fraser et al. [29] deposited on GEO [GSE59027]. The Hi-C matrices for each cell type were downloaded at 100 KB resolution and read into HiCcompare. We performed three comparisons between the cell types, ESC vs. NPC, NPC vs. neuron, and ESC vs. neuron. In each comparison, the data were normalized, low average expression interactions were filtered out, and the differences between the cell types were detected. We also performed a functional enrichment analysis of genes located in differentially interacting regions.

As expected, the ESC vs. neuron had the largest number of differentially interacting regions at 951 (FDR < 0.05). The ESC and NPC had 279 differentially interacting regions, and the NPC and neuron had only 127 differentially interacting regions. These differences expectedly suggest that the undifferentiated ESCs and fully differentiates neuronal cells have many chromatin interaction differences, while the intermediate neural progenitor cells have less differences when compared with either ESCs or neuron cells. These observations suggest that the chromatin structure plays a key role in the process of cell differentiation.

The enrichment analysis for the ESC vs. the neuron found genes enriched in protein binding function, ion channel regulator activity, and “Axon guidance” pathway among others (Additional file 2). The enrichment of these pathways outlines the ESC-to-neuron differentiation processes that are governed by changes in the 3D structure of the genome. When comparing the ESC and NPC cells, genes were found to be enriched in voltage-gated calcium channel activity, ion transporters, and serotonin metabolic processes (Additional file 3). The enrichment results between the NPC and neuron had fewer results but included IgG receptor activity and binding and cytoskeletal protein binding (Additional file 4). These results indicate that the changes in the chromatin structure contain functionally relevant genes for the cell differentiation process.

The results of this HiCcompare analysis show that our methods are capable of detecting biologically meaningful differences in chromatin conformation when comparing different cell types. Together with the results of Fraser et al. [29], the HiCcompare results indicate that the cellular differentiation process involves structural changes of the chromatin, likely leading to the changes in gene expression and the associated biological pathways.

### Comparison with diffHiC

The diffHiC pipeline was designed to process raw Hi-C sequencing datasets and detect chromatin interaction differences using the generalized linear model framework developed in the edgeR package [25]. We compared the results of Hi-C data analyzed in the diffHiC paper (human prostate epithelial cells RWPE1 over-expressing the EGR protein or GFP [18]) with the results obtained by HiCcompare. Because diffHic takes unaligned Hi-C data as input it was not possible to directly compare our method to diffHic using introduced known changes. An additional point to consider for the use of diffHic is that since it is based on the negative binomial GLM methods of edgeR, it requires replicates (or multiple samples per condition) in order to more accurately estimate the negative binomial dispersion parameter. Due to the high costs and relative newness of Hi-C technology, many public datasets do not have any (or very few) replicates thus hampering the estimation of the dispersion factor.

To compare HiCcompare with diffHic we performed a HiCcompare analysis on the RWPE1 Hi-C data [18]. This was compared to the analysis performed in the diffHic paper [25]. We performed the analysis at a 1 MB resolution as described in the diffHic paper. diffHic detected a total of 5737 significant differences (FDR < 0.05), while HiCcompare tended to be more conservative, detecting 680 differences (FDR < 0.05) and 5215 differences when multiple testing correction was not applied (*p*-value < 0.05). Of the 680 differences, 208 overlapped with the regions detected by diffHic. Surprisingly, although diffHiC used CNV correction in their analysis, 2567 (44.7%) of the detected differentially interacting regions overlapped with CNV regions detected in our analysis, and/or blacklisted regions. diffHic tended to detect differentially interacting regions with smaller fold changes as compared to HiCcompare, and at shorter distances between interacting regions, while HiCcompare can detect differences across the full range of distances (Section 6, Additional file 1). These results suggest that detecting chromatin interaction differences represented in the MD coordinates, as implemented in HiCcompare, may be useful in detecting large chromatin interaction differences across the full range of distances, potentially having a more significant biological effect.

### Comparison with FIND

The recently published FIND tool uses a spatial Poisson process to detect differences between two Hi-C experimental conditions [30]. FIND is presented as a tool for high-resolution Hi-C data and treats interactions as spatially dependent on surrounding interactions. In order to compare HiCcompare with FIND, we performed a comparative analysis between Hi-C data from K562 and GM12878 cells lines (Section 7, Additional file 1) as done in the FIND paper [30]. The maximum resolution of each Hi-C matrix was calculated using the calculate_map_resolution.sh function from Juicer [31]. Briefly, two replicates for each cell line were obtained (see Methods), and the replicate contact matrices were combined for the HiCcompare analysis. HiCcompare was used to jointly normalize the data between the cell lines and then detect differences. HiCcompare analyses were performed at 1 MB, 100 KB, 50 KB, 10 KB, and 5 KB resolutions. Additionally, the analyses of GM12878 and K562 were used to compare the run times of HiCcompare and FIND (Section 7, Additional file 1).

The number of differences detected by HiCcompare at 5 KB resolution was much lower than the number FIND detected (~ 150,000) [30]. The drop off of the number of differential interactions detected at high resolution by HiCcompare can be explained by the sparsity and the limited dynamic range of interaction frequencies at 5 KB resolution. Additionally, the large number of differences detected by FIND at 5 KB resolution are questionable given that the maximum resolution of the K562 and GM12878 data was found to be ~ 39 KB and ~ 9 KB, respectively (Section 7, Additional File 1).

The differentially interacting regions detect by HiCcompare at different resolutions were intersected with gene locations, and a KEGG pathway enrichment analysis was performed. The enrichment analysis showed that many of the differential regions contained genes involved in the immune system (Table 2). We also found that the enrichment analyses of HiCcompare-detected differences at each resolution were relatively consistent further indicating the strength of HiCcompare at detecting biologically relevant differences across data resolutions. Despite the differences in resolution of data used for differential analysis (5 kb for FIND and 50 kb - 1 Mb for HiCcompare) the enrichment analysis of HiCcompare-detected differences identified pathways related to the immune system, similar to the results of the FIND analysis. These observations suggest that both methods can detect biologically significant differences.

**Table 2:** Gene enrichment results for HiCcompare analyses

Pathway	1 MB	100 KB	50 KB
Systemic lupus erythematosus	3.807e-06	6.302e-17	1.025e-02
Antigen processing and presentation	3.807e-06	6.808e-01	9.974e-01
*Staphylococcus aureus* infection	8.170e-03	2.354e-01	7.604e-01
Viral myocarditis	8.170e-03	1.038e-01	9.657e-01
Allograft rejection	8.170e-03	1.518e-01	9.974e-01
Viral carcinogenesis	3.327e-02	3.659e-08	3.273e-01
Pathways in cancer	9.162e-01	2.236e-02	9.409e-01

KEGG pathways and their corresponding FDR-corrected p-values for the enrichment analyses of HiCcompare-detected differences at 1 MB, 100 KB, and 50 KB resolutions. Differentially interacting regions detected by HiCcompare were intersected with gene locations, and the overlapping genes were tested for enrichment using EnrichR [37]

To compare the performance of FIND and HiCcompare when a priori known differences were introduced we used replicated data for GM12878 cells. The GM12878 replicates are expected to contain minimal differences, thus suitable for introducing a priori controlled changes and applying both tools in order to detect them. For the data to be entered into FIND, we used the VC squared normalization method from Juicer as described in the FIND paper and the raw data was entered into HiCcompare. We performed this analysis at a resolution of 1 MB (we encountered issues due to extremely long run times of FIND when attempting comparisons at higher resolutions) with fold changes of 2, 3, and 5 for the true changes. HiCcompare successfully detected the majority of the controlled changes while FIND detected smaller differences and was missing most of the introduced controlled changes (Section 7, Additional File 1). Additionally, we found that the run time of FIND on Hi-C matrices at resolutions between 100 KB and 10 KB was extremely long (> 72 h) even when run in parallel on 16 cores, while HiCcompare was able to complete an analysis within minutes (Additional file 1: Figure 3.1). These results further strengthen the notion that HiCcompare detects large chromatin interaction differences potentially having a larger biological impact on genome structure, and does it across the full range of distances.

### Preservation of A/B compartments

A/B compartments are the best known genomic structures that can be detected from Hi-C data [6]. To understand the consequences of the joint vs. individual normalization methods on the detection of A/B compartments we compared principal components defining compartments in raw vs. normalized data. The concordance of compartment detection was evaluated using three metrics: 1) the Pearson correlation coefficient between the vectors of principal components (PCs) detected from raw and normalized data, 2) the overlap of signs of PCs defining A (positive) and B (negative) compartments, and 3) the Jaccard overlap statistics. A/B compartments detected following joint normalization were the most similar to those detected in the raw data (Table 3). These results suggest that the joint HiCcompare normalization preserves properties of Hi-C data needed for the accurate detection of A/B compartments.

**Table 3:** Similarity between A/B compartments detected following various normalization methods

Comparison	Mean Absolute Correlation	Mean Percentage	Jaccard A	Jaccard B
Loess vs. Raw	0.9954	0.8537	0.7971	0.7823
MA vs. Raw	0.9950	0.8539	0.7881	0.7706
ICE vs. Raw	0.9795	0.7850	0.6731	0.6277
KR vs. Raw	0.9489	0.7771	0.5945	0.5000
SCN vs. Raw	0.9309	0.8083	0.6134	0.5495
ChromoR vs. Raw	0.8093	0.6810	0.5210	0.4803

“Correlation” - Pearson correlation coefficient between principal components defining A/B compartments in raw vs. normalized Hi-C data; “Prop. Match Sign” - the proportion of regions with matching signs defining A/B compartments; “Jaccard A/B” - Jaccard overlap statistics between A/B compartments, respectively. All values represent averages over all chromosomes

### Summary and future directions

HiCcompare can be used to compare processed Hi-C libraries between two biological conditions. HiCcompare represents a user-friendly method for the scientific community to begin analyzing the differences in the 3D genome while making use of publicly available datasets. HiCcompare can also easily be integrated into the existing juicer [31], HiC-Pro [17], and other Hi-C pre-processing pipelines for those generating and analyzing new Hi-C experiments. A future extension of HiCcompare is planned to make use of Hi-C experiments where multiple replicates or samples are available for each group.

## Conclusions

This work introduces three novel concepts for the joint normalization and differential analysis of Hi-C data, implemented in the HiCcompare R package. First, we introduce the representation of the differences between two Hi-C datasets on an MD plot, a modification of the MA plot [22]. Importantly, we consider the data on a per-distance basis, allowing the data-driven normalization of global biases without distorting the relative distribution of interaction frequencies of the interacting regions. Second, we implement a non-parametric loess normalization method that minimizes bias-driven differences between the datasets. There is compelling evidence that non-parametric normalization methods, such as quantile- and loess normalization, are particularly suitable for removing between-dataset biases [32, 33], confirmed by our application of loess to the joint normalization of Hi-C data. Third, we develop and benchmark a simple but rigorous statistical method for the differential analysis of Hi-C datasets.

The importance of joint normalization when comparing Hi-C datasets has been demonstrated using MA normalization introduced in the diffHiC R package [25]. MA normalization uses a similar concept of representing measures from two datasets on a single plot [25], except it uses the **A**verage chromatin interaction frequency as the X-axis instead of the **D**istance. MA normalization performed second to HiCcompare (Table 1 and Section 5, Additional File 1). This may be due to the power-law decay of interaction measures leading to the limited dynamic range of average chromatin interaction frequencies and making fitting a loess curve difficult. Instead, the more balanced representation of chromatin interaction differences **M** (Y-axis) as a function of distance **D** (X-axis) improves the performance of the loess fit for the joint normalization and the subsequent detection of chromatin interaction differences.

The discrepancy of differential chromatin interaction detection between diffHiC and HiCcompare (Section 6, Additional File 1) could arise from multiple factors. diffHiC’s implementation of MA normalization favors differences at shorter distances and small fold changes while HiCcompare’s loess fitting through the MD plot allows for the detection of large chromatin interaction differences across the full range of interaction frequencies (Section 6, Additional File 1). diffHiC operates on log counts per million (logCPM) while HiCcompare uses log interaction frequency counts. diffHiC uses enzyme cut sites to define bins when partitioning the genome while HiCcompare uses fixed bin sizes. diffHiC uses median inter-chromosomal interaction frequency to filter low-abundance bin pairs while HiCcompare filters based on average IFs of the chromosome being considered. Finally, the RWPE1 data analyzed by diffHiC is relatively sparse even at 1 MB resolution, potentially interfering with HiCcompare’s statistical analyses. In summary, diffHiC and HiCcompare may provide complementary views on chromatin interaction differences, with HiCcompare being better suited for removing the between-datasets biases and the detection of biology-driven chromatin interaction differences.

In our comparison with FIND (Section 7, Additional file 1), we found that HiCcompare performed better than FIND on data at resolutions between 1 MB and 10 KB. As most publicly available Hi-C data is too sparse to make meaningful inferences at resolutions greater than this, HiCcompare looks to be the better choice for detecting differences on most currently available data. In the case of extremely high-resolution Hi-C data, FIND may be able to pull out more significant differences between two experimental conditions albeit at the expense of significantly longer run times. Comparing our gene enrichment results for GM12878 vs. K562 with those presented in [30], both methods were able to detect differences in regions involved in the immune system as would be expected to occur for these cell types.

Despite the ability of Hi-C technology to simultaneously capture all genomic interactions, current resolution of Hi-C data (1 MB - 1 KB) remains insufficient to resolve individual *cis*-regulatory elements (~100b-1 KB). Alternative techniques, such as ChiA-PET [34], Capture Hi-C [1] have been designed to identify targeted 3D interactions, e.g., between promoters and distant regions. These data require specialized normalization [35] and differential analysis [36] methods. Our future goals include extending the loess joint normalization method for chromosome conformation capture data other than Hi-C.

## Availability and requirements

HiCcompare is available as an open-source R package on Bioconductor and can be installed using the standard Bioconductor installation procedures as described at https://bioconductor.org/packages/HiCcompare/. The development of HiCcompare can be followed on GitHub at https://github.com/dozmorovlab/HiCcompare. HiCcompare is freely available under the MIT open-source software license. HiCcompare is platform independent, and the only requirements are the R and Bioconductor computing environments.

## Additional files

Additional file 1: Supplementary materials for the paper. This PDF file contains supplemental methods (Section 1), a computation performance evaluation of HiCcompare (Section 3), additional validation of methods used in HiCcompare, and extended comparisons with diffHic and FIND (Section 6 & 7). (PDF 5878 kb)

Additional file 2: Table of gene enrichmend results for ESC vs neuron. This excel file contains a worksheet for the GO MF, GO BP, and KEGG pathway analysis results for the gene enrichment analysis between the ESC and neuron discussed in the results section. (XLSX 46 kb)

Additional file 3: Table of gene enrichment results for ESC vs NPC. This excecl file contains a worksheet for the GO MF, GO BP, and KEGG pathway analysis results for the gene enrichment analysis between the ESC and NPC discussed the in the results section. (XLSX 15 kb)

Additional file 4: Table of gene enrichment results for NPC vs Neuron. This excecl file contains a worksheet for the GO MF results for the gene enrichment analysis between the NPC and Neuron. The GO BP and KEGG pathway analysis did not return any significant results and thus are not included here. (XLSX 11 kb)

## Abbreviations

CNV: Copy Number Variation
ESC: Embryonic stem cells
ICE: Iterative Correction and Eigenvector decomposition
IF: Interaction Frequency
KR: Knight-Ruiz normalization
MA plot: Minus vs Average plot
MCC: Matthews Correlation Coefficient
MD plot: Minus vs Distance plot
NPC: Neural progenitor cells
SCN: Sequential Component Normalization
