# Supplementary material for: HiCcompare: an R-package for joint normalization and comparison of HI-C datasets
Source: BMC Bioinformatics. 2018 Jul 31;19:279. doi: 10.1186/s12859-018-2288-x (PMC6069782; doi:10.1186/s12859-018-2288-x)
Supplement: Supplementary file 1 — Supplementary materials for the paper. This PDF file contains supplemental methods (Section 1), a computation performance evaluation of HiCcompare (Section 3), additional validation of methods used in HiCcompare, and extended comparisons with diffHic and FIND (Section 6 & 7). (PDF 5878 kb) [file 12859_2018_2288_MOESM1_ESM.pdf]

Supplemental Material

Table of Contents

1 Supplemental Methods	2
2 Distance-centric concept of chromatin interaction frequencies	8
3 Performance evaluation of HiCcompare	10
4 Persistence of bias in individually normalized Hi-C matrices	11
5 Extended evaluation of differential chromatin interaction detection	25
6 Comparison with diffHic	29
7 Comparison with FIND	32

1. Supplemental Methods

Data sources

Supplemental Table 1.1. Hi-C data used in the current study.

Cell line	Resolution	Cutting enzyme	URL
GM12878	1kb - 1mb	MboI	ftp://ftp.ncbi.nlm.nih.gov/geo/series/GSE63nnn/GSE63525/suppl/GSE63525_GM12878_insitu_primary_30.hic.gz
GM12878	1kb - 1mb	DpnII	ftp://ftp.ncbi.nlm.nih.gov/geo/series/GSE63nnn/GSE63525/suppl/GSE63525_GM12878_insitu_DpnII_combined_30.hic.gz
K562	1kb - 1mb	MboI	ftp://ftp.ncbi.nlm.nih.gov/geo/series/GSE63nnn/GSE63525/suppl/GSE63525_K562_combined_30.hic.gz
IMR90	1kb - 1mb	MboI	ftp://ftp.ncbi.nlm.nih.gov/geo/series/GSE63nnn/GSE63525/suppl/GSE63525_IMR90_intrachromosomal_contact_matrices.tar.gz
HMEC	1kb - 1mb	MboI	ftp://ftp.ncbi.nlm.nih.gov/geo/series/GSE63nnn/GSE63525/suppl/GSE63525_HMEC_intrachromosomal_contact_matrices.tar.gz
NHEK	1kb - 1mb	MboI	ftp://ftp.ncbi.nlm.nih.gov/geo/series/GSE63nnn/GSE63525/suppl/GSE63525_NHEK_intrachromosomal_contact_matrices.tar.gz
hESC	1mb	HindIII	ftp://cooler.csail.mit.edu/coolers/hg19/Dixon2012-H1hESC-HindIII-allreps-filtered.1000kb.cool
RWPE1	1mb	HindIII	https://www.ncbi.nlm.nih.gov/geo/query/acc.cgi?acc=GSE37752
ESC/NPC/Neuron	50kb - 100kb	HindIII, NcoI	https://www.ncbi.nlm.nih.gov/geo/query/acc.cgi?acc=GSE59027
Hg19 CTCF sites	NA	NA	https://zenodo.org/record/29423#.WwOtf0gvxPZ

Normalization methods for individual Hi-C datasets

Several methods for normalizing individual Hi-C datasets were compared with the `loess` joint normalization method. Here, we briefly describe them.

The `ChromoR` method (Shavit and Lio' 2014) applies the Haar-Fisz Transform (HFT) to decompose a Hi-C contact map. HFT assumes the IFs in the contact map are distributed as a Poisson random variable. After HFT decomposition, wavelet shrinkage methods for Gaussian noise are applied for de-noising. The contact map is then reconstructed with the inverse HFT. The `ChromoR` R package was used to normalize the matrices with the `correctCIM` function.

`ICE` (iterative correction and eigenvector decomposition) normalization (Imakaev et al. 2012) functions by modeling the expected IF_{ij} for every pair of regions *(i,j)* as $E_{ij} = B_i B_j T_{ij}$, where B_i and B_j are the biases and T_{ij} is the true matrix of normalized IFs. The maximum likelihood solution for the biases B_i is obtained by iterative correction. It attempts to make all regions equally visible, and was shown to perform as well as the explicit bias correction method by Yaffe and Tanay (Belton et al. 2012). `ICE` normalization was performed using the `HiTC` R package's `normICE` function.

`KR` (Knight-Ruiz) normalization (Knight and Ruiz 2012) is another "equal visibility" algorithm that balances a square non-negative matrix A by finding a diagonal scaling of A such that $P = D_1 A D_2$ sums to one. The `KR` algorithm uses an iterative process to find D_1 and D_2 scaling matrices by alternately normalizing columns and rows in a sequence of matrices using an approximation of Newton's method. The `KR` normalization method was re-implemented in R using the published `matlab` code (Knight and Ruiz 2012) and is included in the `HiCcompare` package as the `KRnorm` function.

`SCN` (Sequential Component Normalization) (Cournac et al. 2012) is a method that is broadly generalizable to many Hi-C experimental protocols. It attempts to smooth out biases due to GC content and circularization. `SCN` works by first normalizing each column vector of a Hi-C contact matrix to one using the Euclidean norm. Then each row of the resulting matrix is normalized to one using the row Euclidean norm. This process is repeated until convergence (usually 2 to 3 iterations). The SCN method was re-implemented in R and included in the `HiCcompare` package as the `SCN` function.

`MA` (Minus Average normalization) (Lun and Smyth 2015) is a commonly used normalization method for genomic data. It is based on the MA plot where the data is plotted according to the Average log counts (or counts per million) and the log Minus (difference) between the two data sets. A loess model is then fit to this plot and the residuals for the fit can be used to smooth the data sets. MA normalization was implemented in R and included in the `HiCcompare` packages as the `MA_norm` function.

Investigation of the distribution of M

Here we show that the distribution of M is approximately normal and that this holds true for different genomic distances, chromosomes, and resolutions.

Chromosome 1

Using GM12878 replicate datasets for chromosome 1 at 1MB resolution we fit a QQ plot for a normal distribution at distances of 5 and 50 showing that after loess normalization M is roughly normal at different distances.

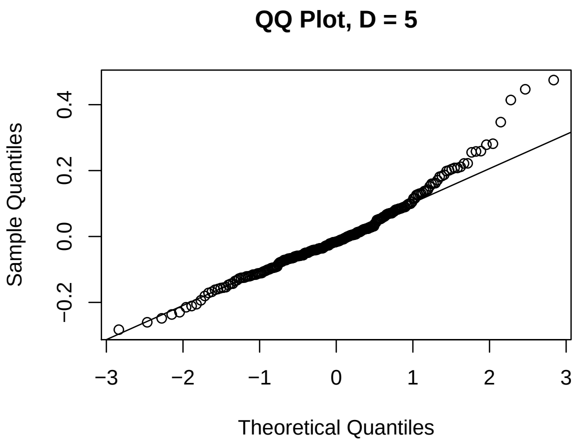

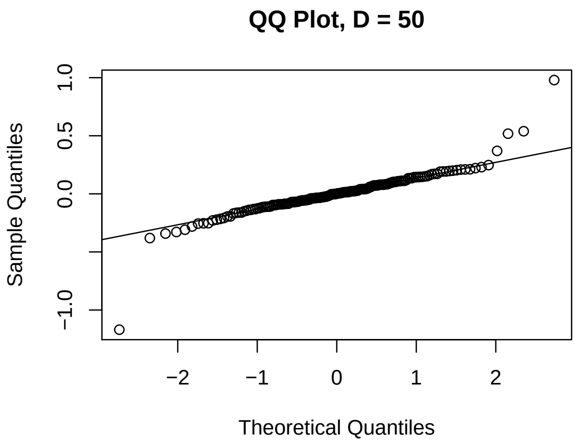

Chromosome 18

Using GM12878 replicate datasets for chromosome 18 at 1MB resolution we fit a QQ plot for a normal distribution at distances of 5 and 50 showing that after loess normalization M is roughly normal at different distances and chromosomes.

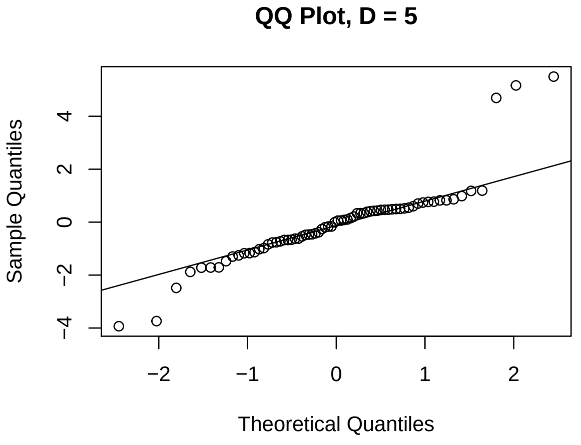

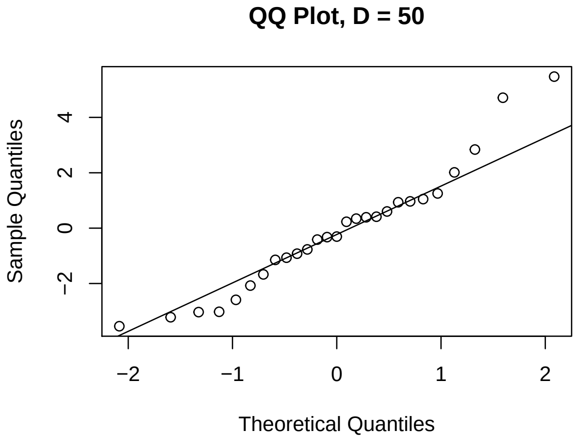

The distribution of M is approximately normal between resolutions

Real Hi-C data from Gm12878 cell line were used. The data used were from chromosome 1 cut either using the DpnII enzyme or MboI enzyme at varying resolutions of 1MB, 500KB, 100KB, 50KB, and 5KB. The increased resolution (smaller length of genomic region) is accompanied by the increased proportion of zero interaction frequencies and the overall smaller dynamic range of IFs.

500KB Resolution

At 500KB resolution we fit a QQ plot for M at distances of 5 and 50.

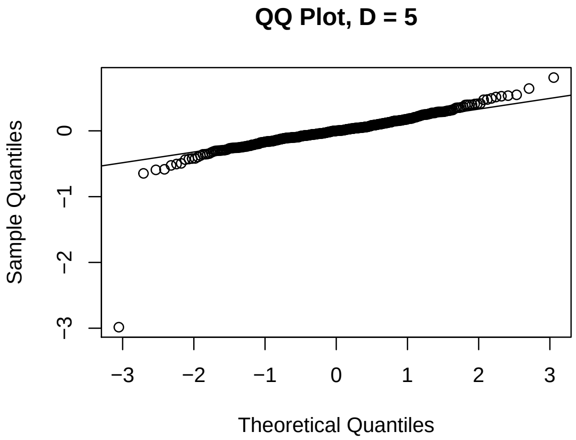

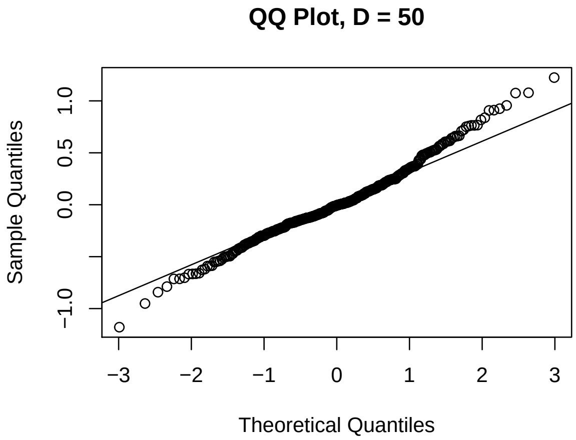

100KB Resolution

At 100KB resolution we fit a QQ plot for M at distances of 5 and 50.

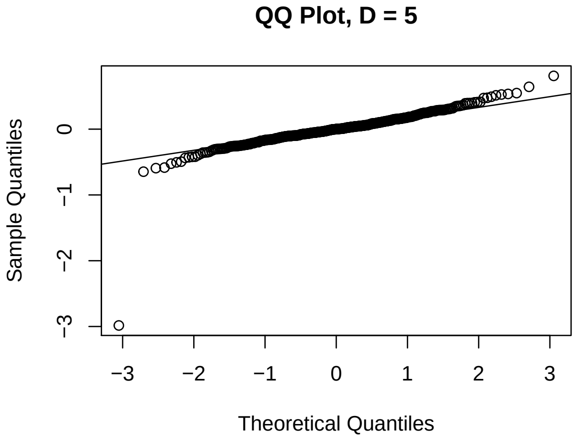

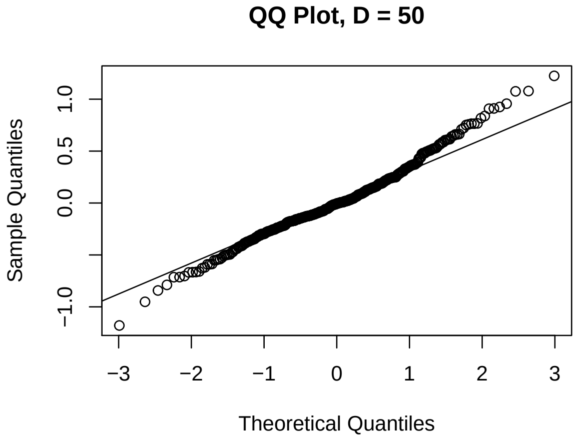

50KB Resolution

At 50KB resolution we fit a QQ plot for M at distances of 5 and 50.

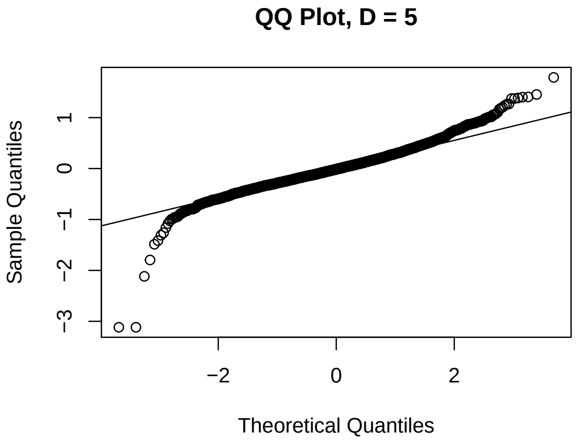

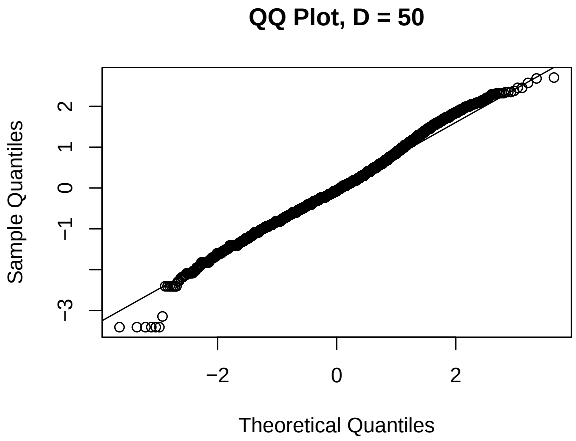

Summary

M has an approximately normal distribution over a range of distances, resolutions, and chromosomes. Thus it is justifiable to convert M values into Z-scores for difference detection. The tails of the M distribution are where the most deviations from the fit to the normal distribution occur. These deviations typically occur for the interactions with low average expression and thus will be filtered out before Z-score conversion.

The distribution of Average Expression A between interacting pairs

Average expression (A) is the mean of IF1 and IF2 where IF1 and IF2 are the Hi-C interaction frequencies for a pair of interacting regions from datasets 1 and dataset 2. Higher values of A indicate that the reads are more trustworthy due to better sequencing coverage. Differences found between interactions with low values of A may not be trustworthy due to the possibility of larger effects of biases, random variation, sequencing errors, etc. Thus it is justifiable to not consider any differences found for interactions with low average expression. A tends to have a very right skewed distribution. This is because interactions coming from closer to the diagonal of a Hi-C matrix tend to have very large IFs (short distance interactions) while the long range interactions tend to have smaller IFs.

The distribution of A over varying resolutions

Here we display the distribution of the Log average expression between GM12878 chromosome 1 data cut with either DpnII or MBOI enzymes at 1MB resolution.

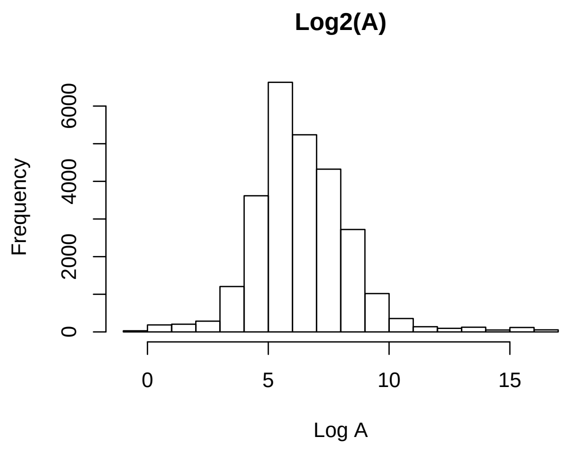

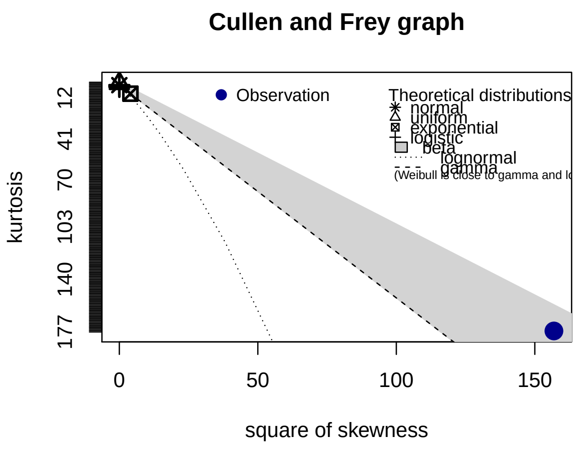


```
summary statistics
------
min:  0.6958433   max:  109875.8
median:  71.43548
mean:  668.1348
estimated sd:  5099.869
estimated skewness:  12.52585
estimated kurtosis:  176.3298
```

Determining which A quantile to filter out

Here we add differences to data from replicates of the GM12878 cell line. We then perform a HiCcompare analysis using a sequence of values for the minimum A value to be filtered out. All differences with an A value less than the minimum value specified are filtered out and ignored. We then plot the number of true positives and false positives against the A value filtered.

There is much greater variability in M at higher resolutions due to the generally lower levels of average expression. The data at 100KB resolution with no changes added to it looks like this on the MD plot after difference detection with no filtering:

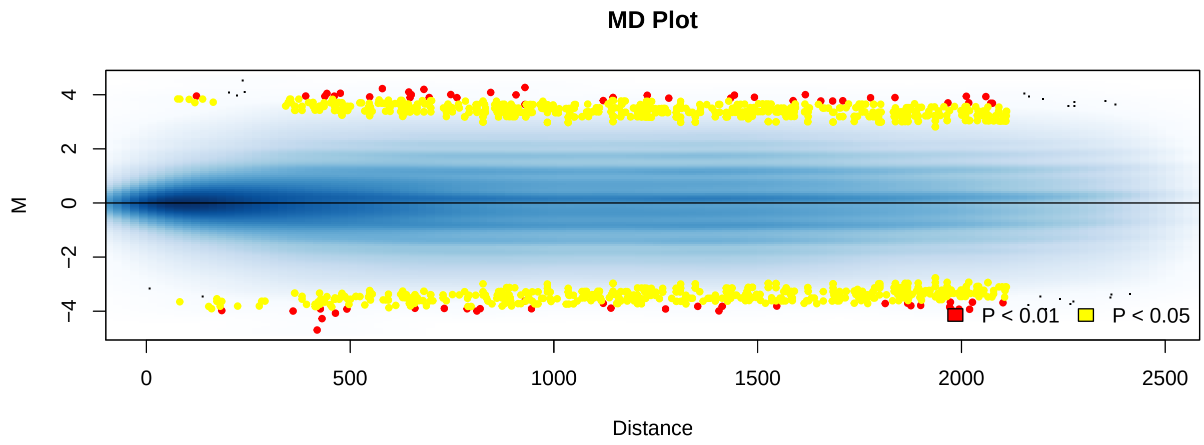

Most of these detected differences have relatively low A values are not very trustworthy. Varying the minimum A filtered and adding in 5,000 true differences at a 4 fold change we get:

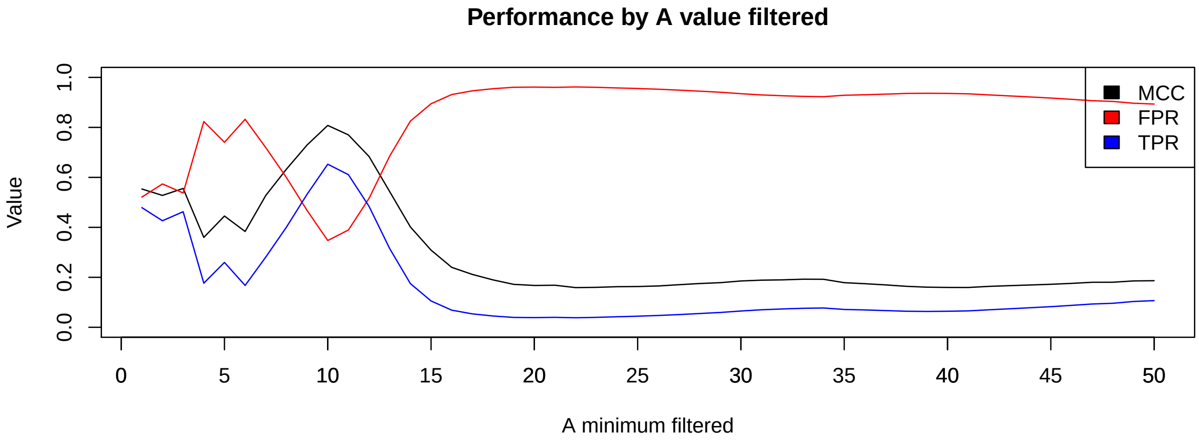

Many of the differences with high M values are derived from interactions where one IF is close to 1 and the other is in the range of 15 to 30. Filtering out any differences where A < 15 gives the best results and allows the most true differences to be detected and the least number of false positives. This may require some tuning to different datasets and resolutions.

Summary

A has a right skewed distribution. Many of the differences detected with low corresponding average expression are not as trustworthy as differences with large values of A. Filtering is required to remove these low A differences. Filtering can be accomplished either using the quantile of A or by setting a minimum acceptable value of A. These difference approaches may work better in some situations than others. Both options are available to the user in `HiCcompare`.

References

Belton J-M, McCord RP, Gibcus JH, Naumova N, Zhan Y, Dekker J. 2012. Hi-c: A comprehensive technique to capture the conformation of genomes. *Methods* **58**: 268–76.

Cournac A, Marie-Nelly H, Marbouty M, Koszul R, Mozziconacci J. 2012. Normalization of a chromosomal contact map. *BMC Genomics* **13**: 436.

Imakaev M, Fudenberg G, McCord RP, Naumova N, Goloborodko A, Lajoie BR, Dekker J, Mirny LA. 2012. Iterative correction of hi-c data reveals hallmarks of chromosome organization. *Nat Methods* **9**: 999–1003.

Knight PA, Ruiz D. 2012. A fast algorithm for matrix balancing. *IMA Journal of Numerical Analysis* drs019.

Lun ATL, Smyth GK. 2015. DiffHic: A bioconductor package to detect differential genomic interactions in hi-c data. *BMC Bioinformatics* **16**: 258.

Shavit Y, Lio' P. 2014. Combining a wavelet change point and the bayes factor for analysing chromosomal interaction data. *Mol Biosyst* **10**: 1576–85.

2. Distance-centric concept of chromatin interaction frequencies

Supplemental Figure 2.1. Non-linear dependence of chromatin interaction frequency vs. distance. The decay of interaction frequencies with distance has been modeled with power-law (Lieberman-Aiden et al. 2009; Sanborn et al. 2015), double exponential (Tanizawa et al. 2010), binomial (Mifsud et al. 2017), Poisson and negative binomial (Jin et al. 2013; Cairns et al. 2016; Hu et al. 2012; Shavit and Lio' 2014), and zero-inflated negative binomial (Di Stefano et al. 2016) distributions. The aforementioned publications acknowledge deviation from the ideal power-law relationship (straight lines). Curved lines represent chromosome-specific `loess` fits of the relationship, colored by dataset. The full range of genomic distances is shown. Data from HMEC, IMR90, NHEK cell lines, using all chromosomes, 500kb resolution were used.

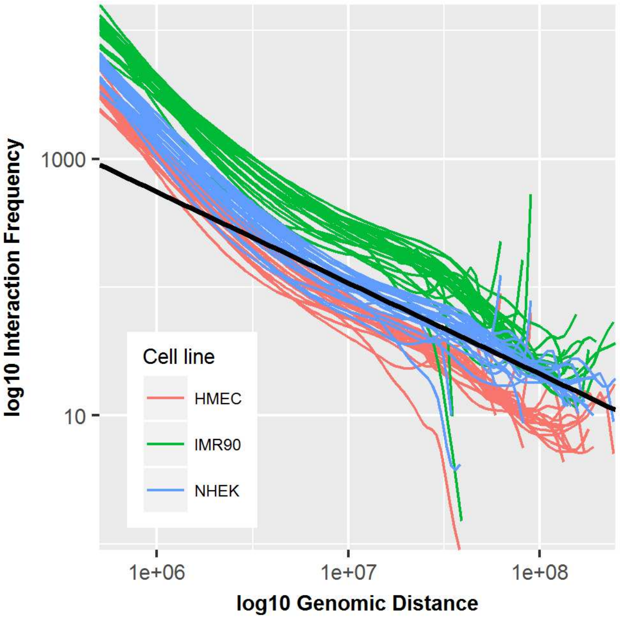

References

Cairns J, Freire-Pritchett P, Wingett SW, Várnai C, Dimond A, Plagnol V, Zerbino D, Schoenfelder S, Javierre B-M, Osborne C, et al. 2016. CHiCAGO: Robust detection of dna looping interactions in capture hi-c data. *Genome Biol* **17**: 127.

Di Stefano M, Paulsen J, Lien TG, Hovig E, Micheletti C. 2016. Hi-c-constrained physical models of human chromosomes recover functionally-related properties of genome organization. *Sci Rep* **6**: 35985.

Hu M, Deng K, Selvaraj S, Qin Z, Ren B, Liu JS. 2012. HiCNorm: Removing biases in hi-c data via poisson regression. *Bioinformatics* **28**: 3131–3.

Jin F, Li Y, Dixon JR, Selvaraj S, Ye Z, Lee AY, Yen C-A, Schmitt AD, Espinoza CA, Ren B. 2013. A high-resolution map of the three-dimensional chromatin interactome in human cells. *Nature* **503**: 290–4.

Lieberman-Aiden E, Berkum NL van, Williams L, Imakaev M, Ragoczy T, Telling A, Amit I, Lajoie BR, Sabo PJ, Dorschner MO, et al. 2009. Comprehensive mapping of long-range interactions reveals folding principles of the human genome. *Science* **326**: 289–93.

Mifsud B, Martincorena I, Darbo E, Sugar R, Schoenfelder S, Fraser P, Luscombe NM. 2017. GOTHiC, a probabilistic model to resolve complex biases and to identify real interactions in hi-c data. *PLoS One* **12**: e0174744.

Sanborn AL, Rao SSP, Huang S-C, Durand NC, Huntley MH, Jewett AI, Bochkov ID, Chinnappan D,

Cutkosky A, Li J, et al. 2015. Chromatin extrusion explains key features of loop and domain formation in wild-type and engineered genomes. *Proc Natl Acad Sci U S A* **112**: E6456–65.

Shavit Y, Lio' P. 2014. Combining a wavelet change point and the bayes factor for analysing chromosomal interaction data. *Mol Biosyst* **10**: 1576–85.

Tanizawa H, Iwasaki O, Tanaka A, Capizzi JR, Wickramasinghe P, Lee M, Fu Z, Noma K-i. 2010. Mapping of long-range associations throughout the fission yeast genome reveals global genome organization linked to transcriptional regulation. *Nucleic Acids Res* **38**: 8164–77.

3. Performance evaluation

Supplemental Figure 3.1. Effect of parallelization on HiCcompare runtime Run time (Y-axis) of `HiCcompare` normalization using 1, 4, 8 and 12 1596 MHz cores (X-axis) with 10gb of memory on CentOS 6.8 operating system. Time to normalize two RWPE datasets (see Methods) over chromosomes 1-22 was averaged over $n = 10$ runs. Data at 1Mb, 500kb, 100kb, 50kb (color legend) was used, expectedly requiring less/more run time, respectively.

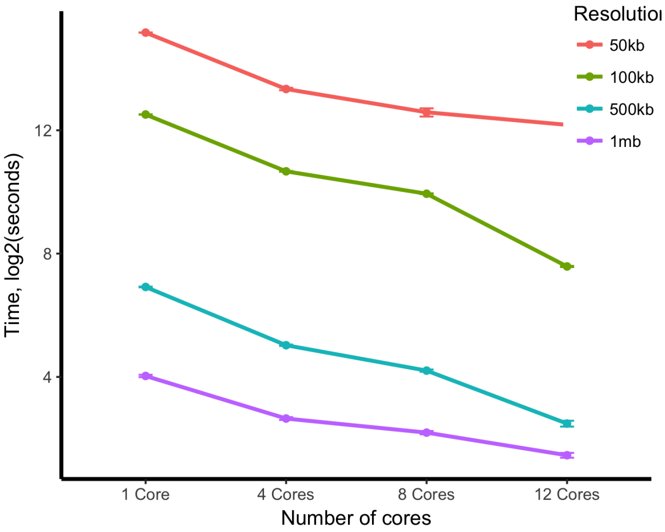

4. Persistence of bias in individually normalized chromatin interaction matrices, and its effect on the detection of differential chromatin interactions

Introduction

To compare the ability of methods for normalizing individual datasets to remove biases *between* chromatin interaction matrices, we compare individually normalized matrices with the jointly normalized ones. Several parameters were assessed:

- The effect of global differences. Most of the time different chromatin interaction matrices will contain different total numbers of reads, resulting in overall differences. We assessed whether methods for normalizing individual datasets were able to account for the differences in the total number of reads.
- The ability of the joint normalization to account for biases under different conditions, such as when comparing matrices obtained with different cutting enzymes, or matrices from different chromosomes.
- The effect of individual and joint normalization methods on detecting chromatin interaction differences.

Investigation of the `loess` joint normalization over varying resolutions

Hi-C data from Gm12878 cell line were used. The data used were from chromosome 1 cut either using the DpnII enzyme or MboI enzyme at varying resolutions of 1MB, 500KB, 100KB, 50KB, and 5KB. The increased resolution (smaller length of genomic region) is accompanied by the increased proportion of zero interaction frequencies and the overall smaller dynamic range of IFs. The goal of this section is to observe the effect of resolution on the performance of joint `loess` normalization.

Here the `hic_loess` procedure is performed for the comparison of MboI and DpnII in GM12878 for chromosome 1 at varying resolutions.

1MB

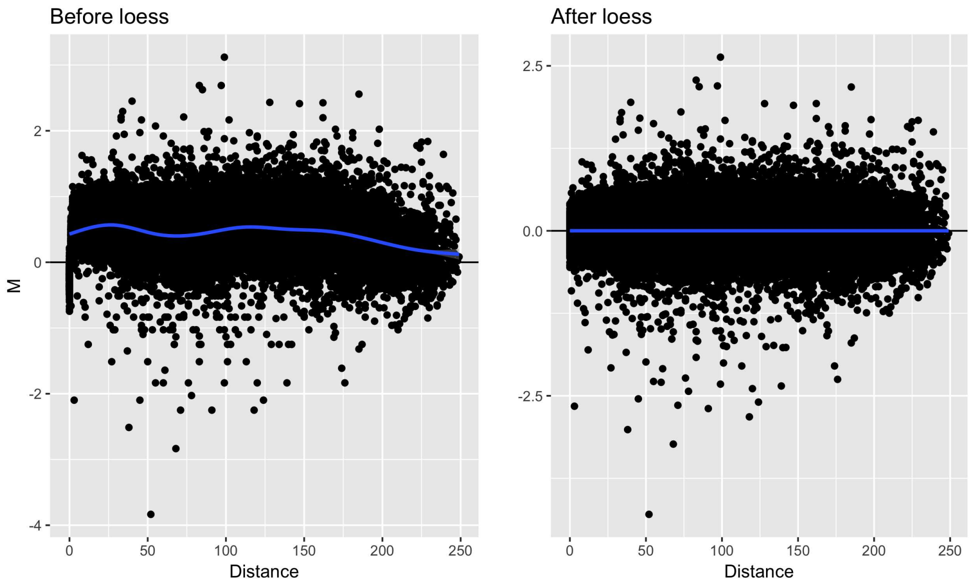

500KB

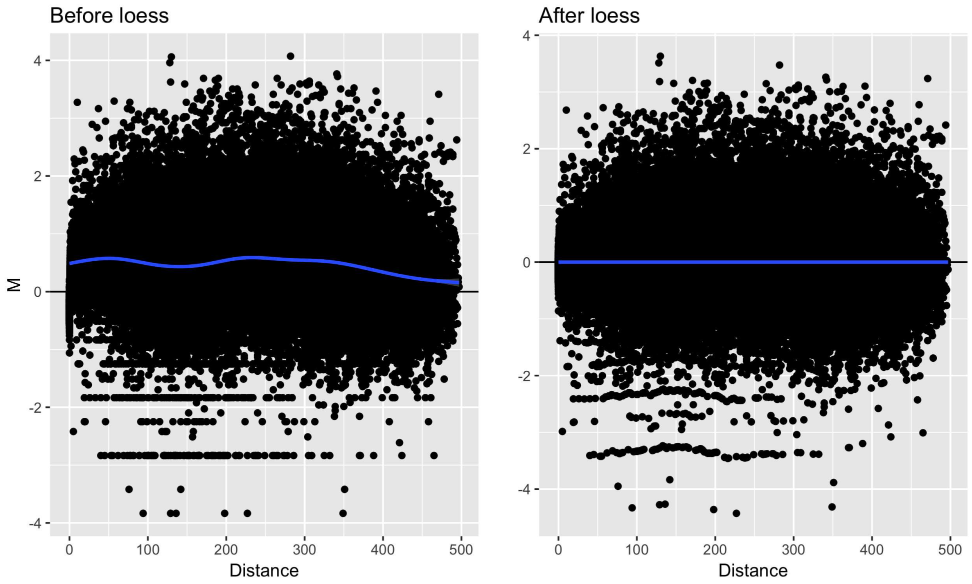

Before loess
After loess
M
Distance
Distance
4
2
0
-2
-4
0
100
200
300
400
500

100KB

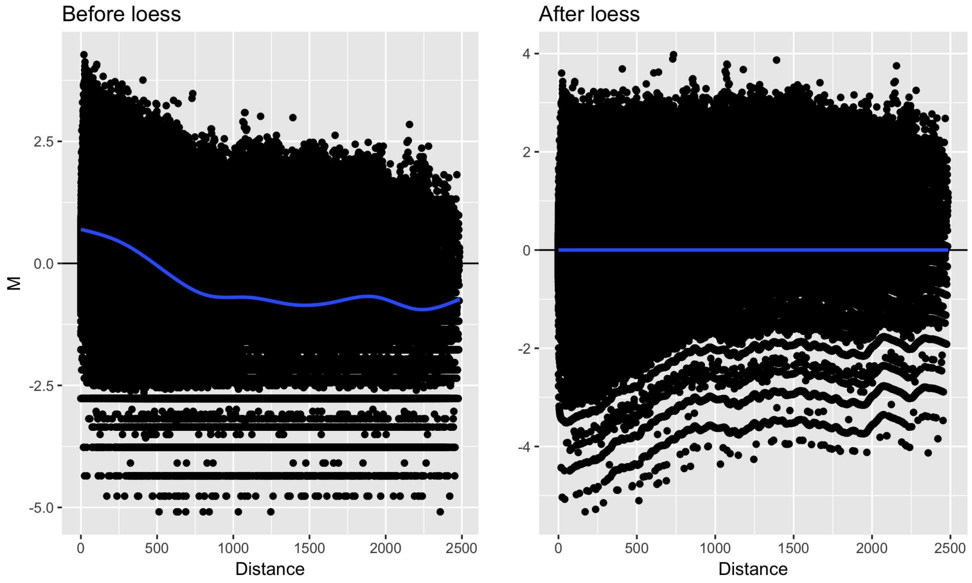

Before loess
After loess
M
Distance
Distance
2.5
0.0
-2.5
-5.0
4
2
0
-2
-4
0
500
1000
1500
2000
2500

50KB

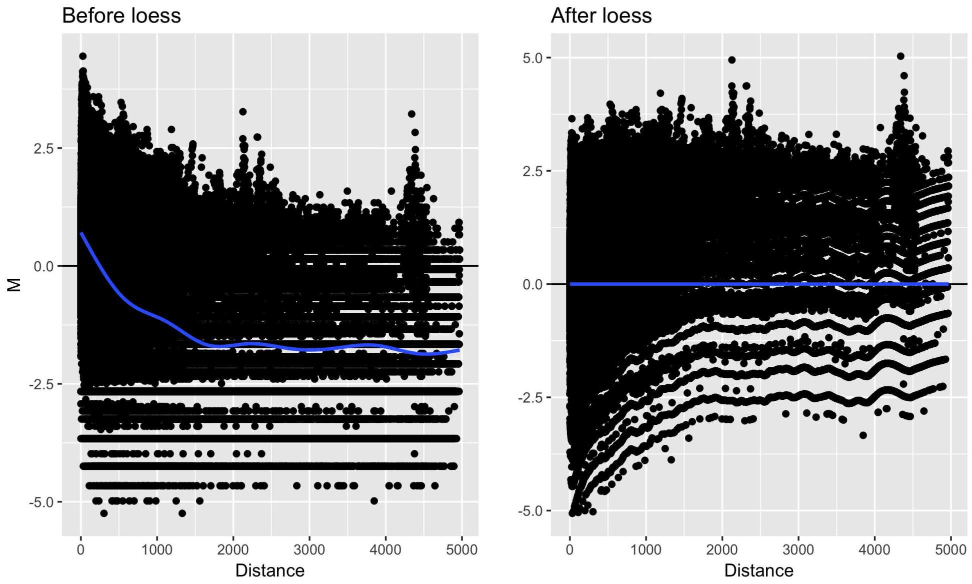

Summary

`loess` works well for removing biases at resolutions between 1MB and 50KB. Once the resolution is higher than 50KB, the procedure begins to fail due to the sparsity of the data. At high resolutions Hi-C data becomes very sparse with most values in the matrix being 0 or a small number. Thus when plotted on the MD plot the sparsity begins to show as the straight horizontal lines of points representing very small differences existing between the two datasets due to the sparsity of the sequencing coverage. As sequencing techniques improve and the depth of Hi-C sequencing is increased the issues of sparsity at higher resolutions should lessen.

The effect of normalization methods on removing global differences

Hi-C matrices may have a different total number of reads. This imbalance will lead to the overall difference between the two matrices, reflected by the global shift of the cloud of M differences from zero. The unscaled matrices, globally shifted from $M = 0$, can be successfully normalized by `loess`. However, individually normalized matrices will still contain the global shift, as shown below. By default, the `create.hic.table` function rescales the matrices to have the same total number of reads. Rescaling is accomplished by first calculating the scale factor $\psi = \frac{\sum IF_i}{\sum IF_j}$ where i is the set of all the IFs for the upper triangle of the first Hi-C matrix and j is the set of all IFs for the upper triangle of the second Hi-C matrix. Next, IF_j is scaled by setting $IF_{j_{new}} = \frac{IF_j}{\psi}$.

`loess`

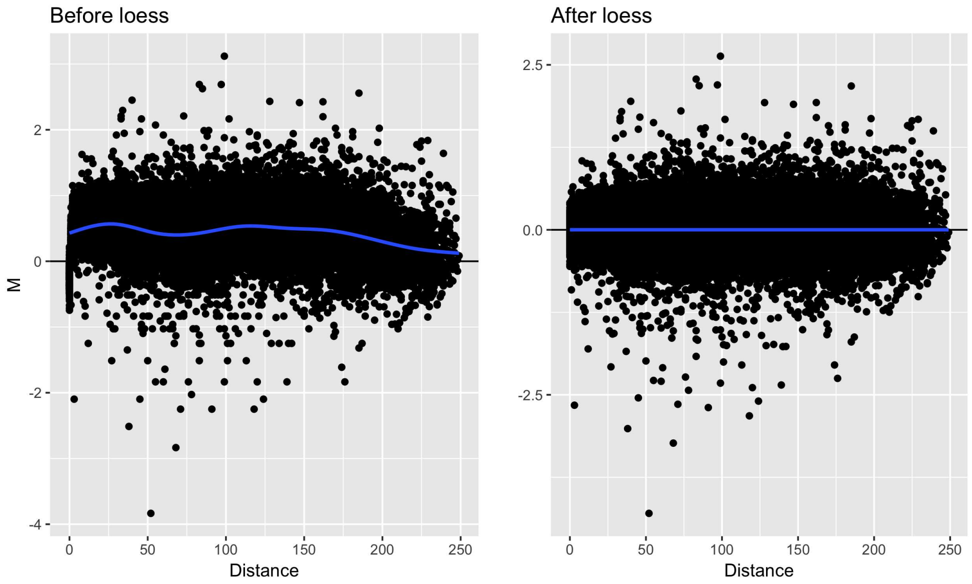

`ChromoR`

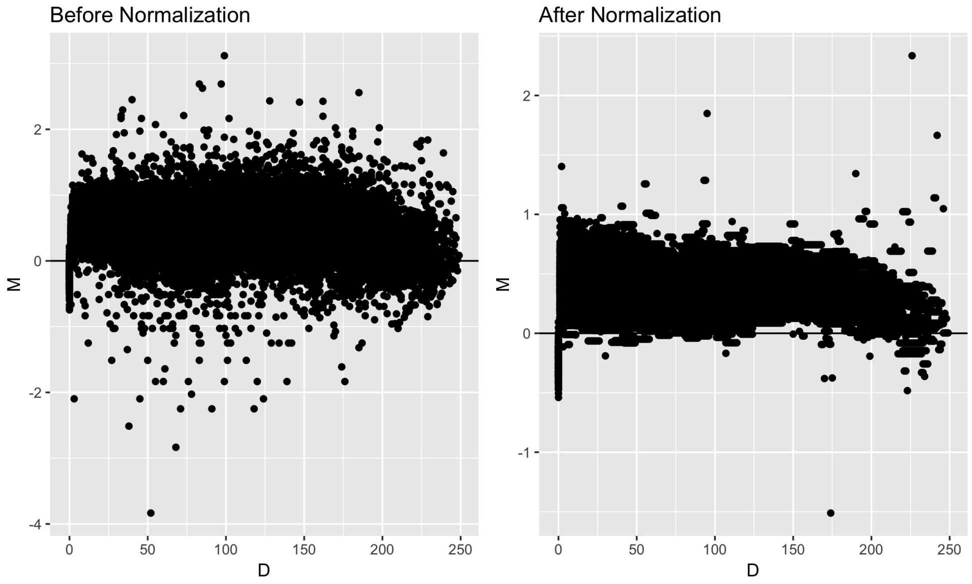

ICE

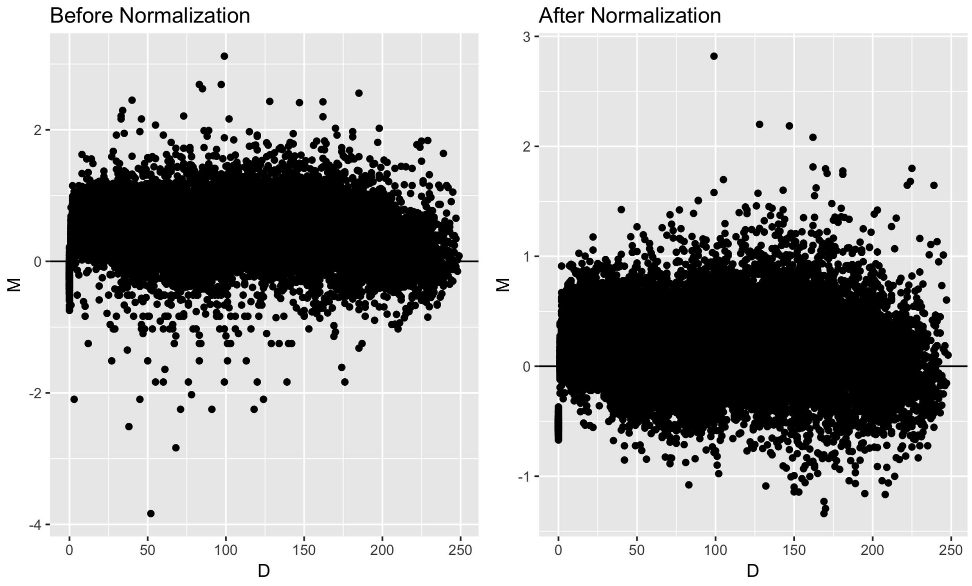
Before Normalization
After Normalization
M
D
2
0
-2
-4
3
1
-1
0
50
100
150
200
250

KR

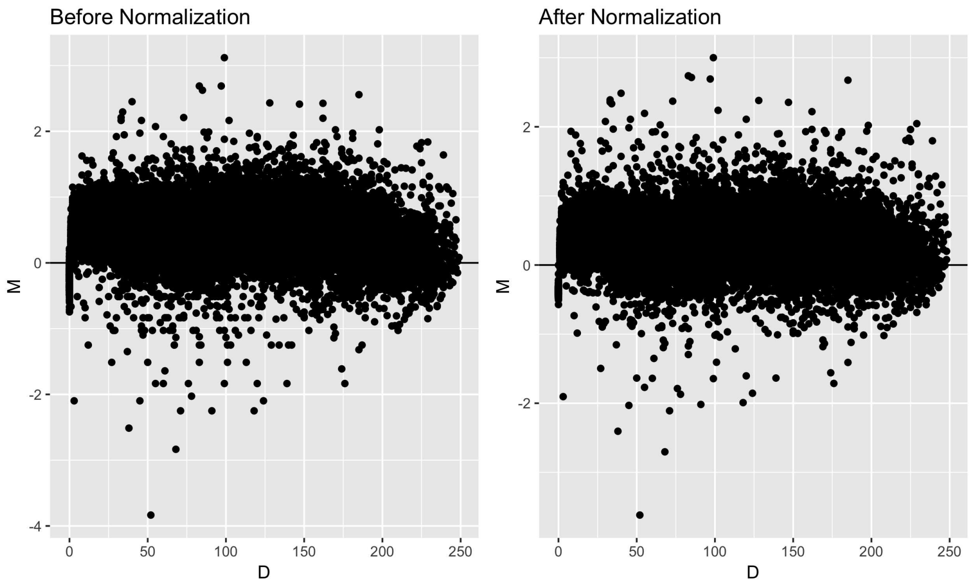
Before Normalization
After Normalization
M
D
2
0
-2
-4
0
50
100
150
200
250

SCN

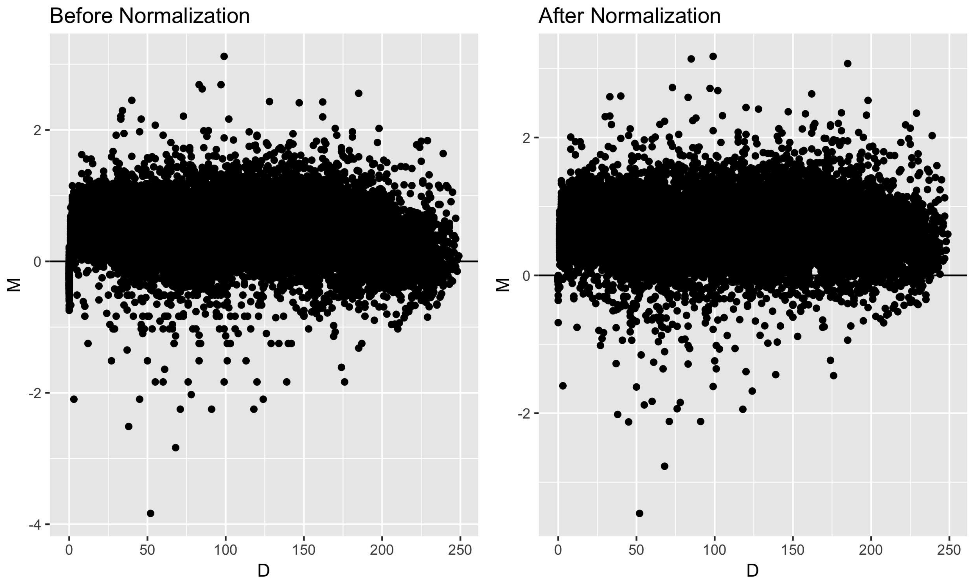

Summary

As can be seen from the above, the MD plots for the single matrix normalization methods do not all succeed at re-scaling the data and thus the main cloud of points are not centered around $M = 0$. `Loess` however, was able to take care of re-scaling the data and centered the MD plot around 0. Global scaling is recommended for any comparisons between Hi-C datasets.

The effect of `loess` normalization on removing chromosome-specific biases

Common cutting enzyme

The MD plot below displays data before and after joint `loess` normalization from GM12878 at 1MB resolution, chromosome 1, that were obtained as replicates using the same cutting enzyme. Since the data here is replicate data it is expected that there will not be many differences between the datasets. Any differences found are assumed to be due to bias in the sequencing procedures.

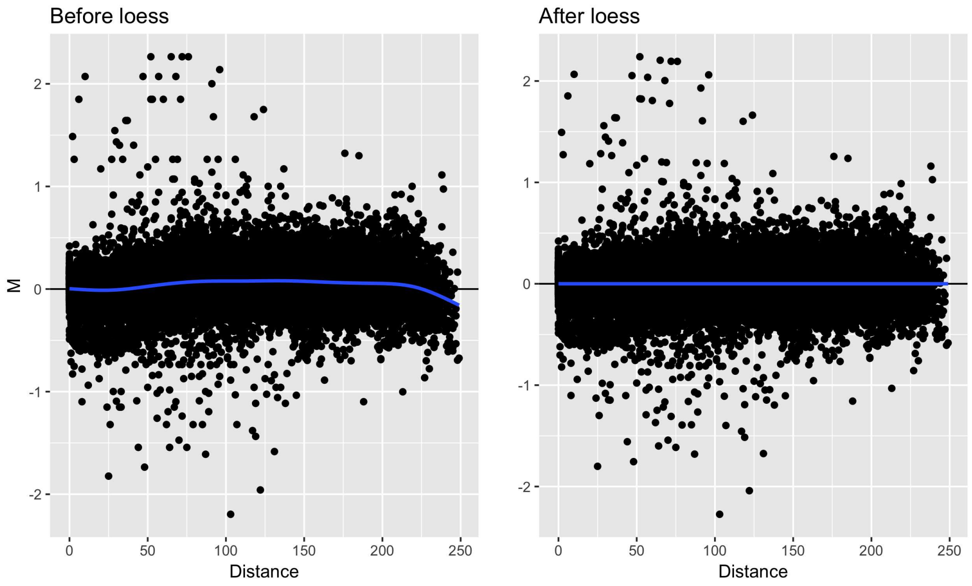

As can be seen by the loess fit on the "Before loess" MD plot there is not a large amount of bias between the two datasets.

Different cutting enzymes

The Hi-C datasets here are from GM12878 cell lines at 1MB resolution, chromosome 1. One dataset was cut using MboI and the other using DpnII. Since different cutting enzymes are used it is expected that there will be some differences in the data due to enzyme choice. Biases between the datasets are successfully removed with `loess` normalization as can be seen in the following MD plots displaying the data before and after joint `loess` normalization. It can also be seen that biases between the datasets differ between each chromosome and dataset. The differences do not appear to follow a trend which makes a non-parametric approach to normalization better suited to the task.

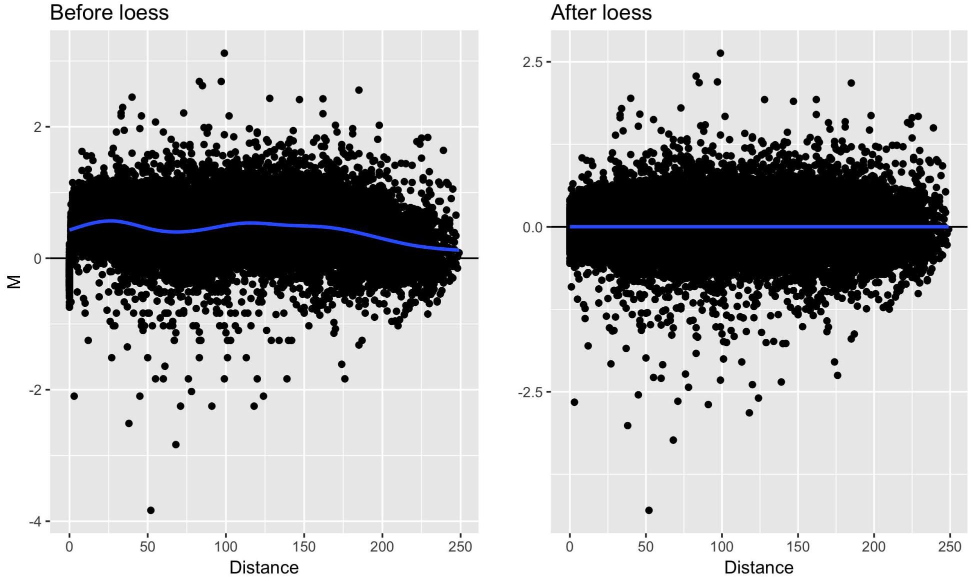

The effect of normalization methods on detecting differential chromatin interactions

To look at differences between different normalization methods we use data from GM12878 at 1MB resolution on chr 11 generated using two different cutting enzymes, MboI and DpnII. The data is scaled. For each method tested below, we also test for differences between the two datasets. No artificial changes were added to the datasets. Any differences detected by the method will be examples of existing differences between the replicated Hi-C data on the same cell line when cut by different enzymes. Since the datasets are for the same chromosome and the same cell line we should expect few differences to be detected.

`loess`

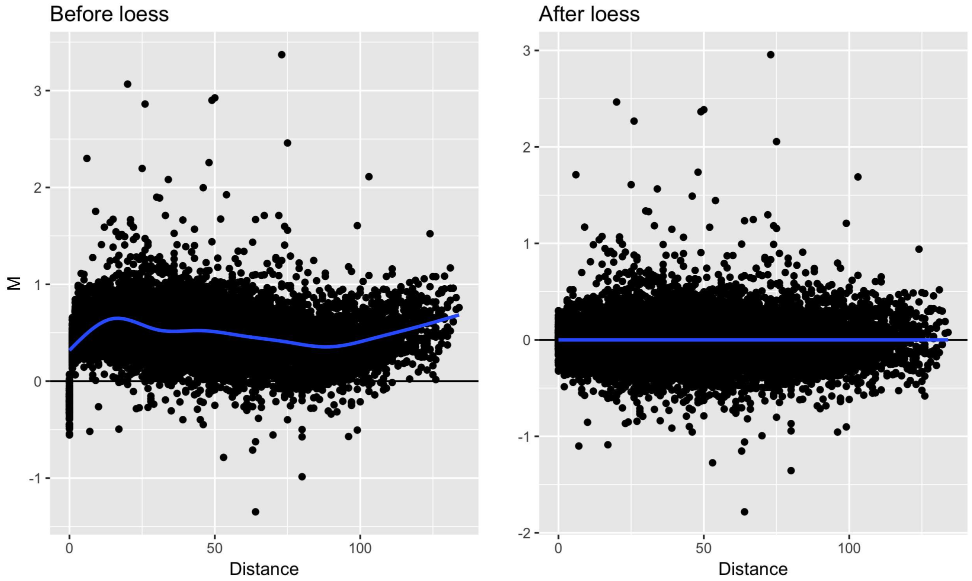

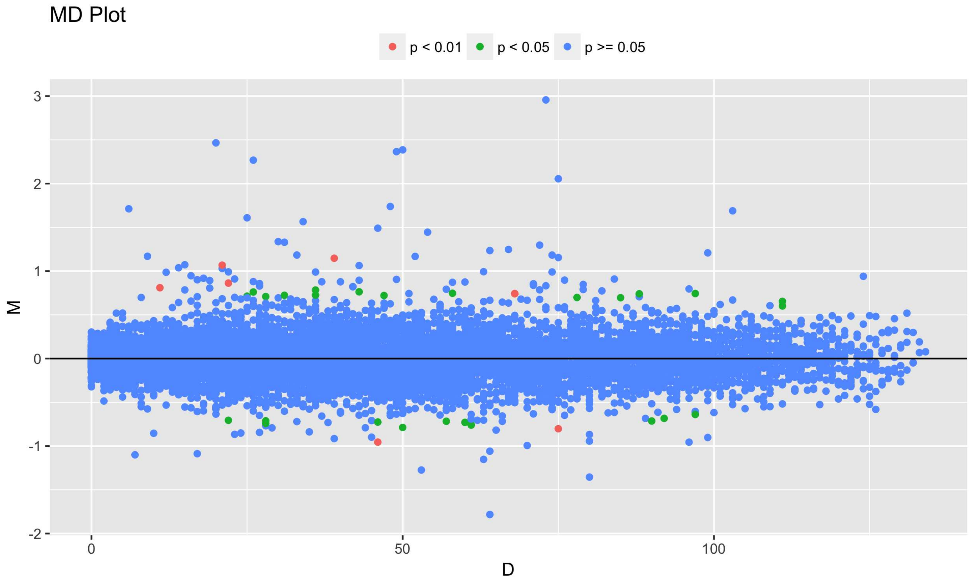


```
[1] "39 differences found between the datasets"
```

The MD plot above serves as a reference to show that `Loess` can successfully normalizes the data and removes bias between the two datasets. The following MD plots display the data after the specified individual

normalization method has been applied to each matrix.

`ChromoR`

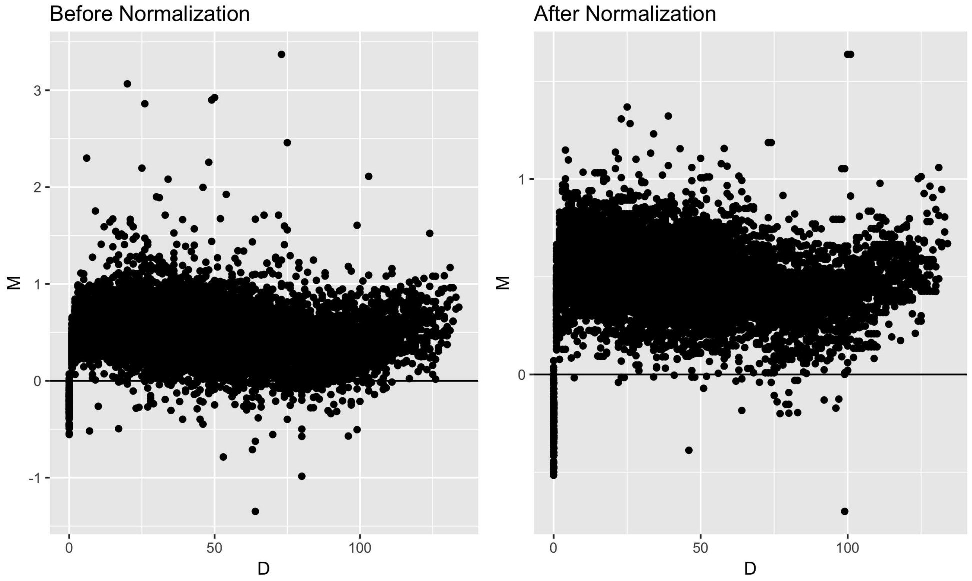

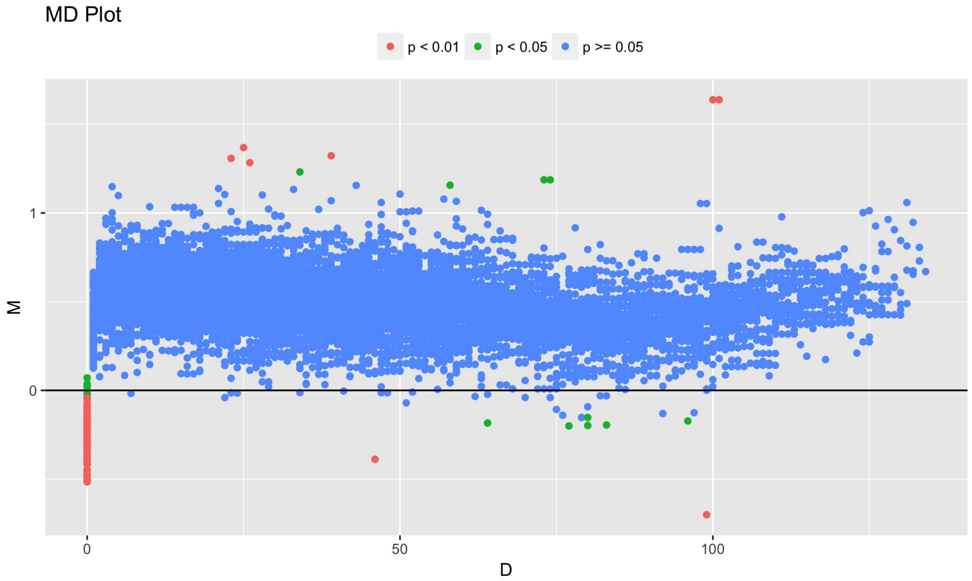


```
[1] "153 differences found between the datasets"
```

ICE

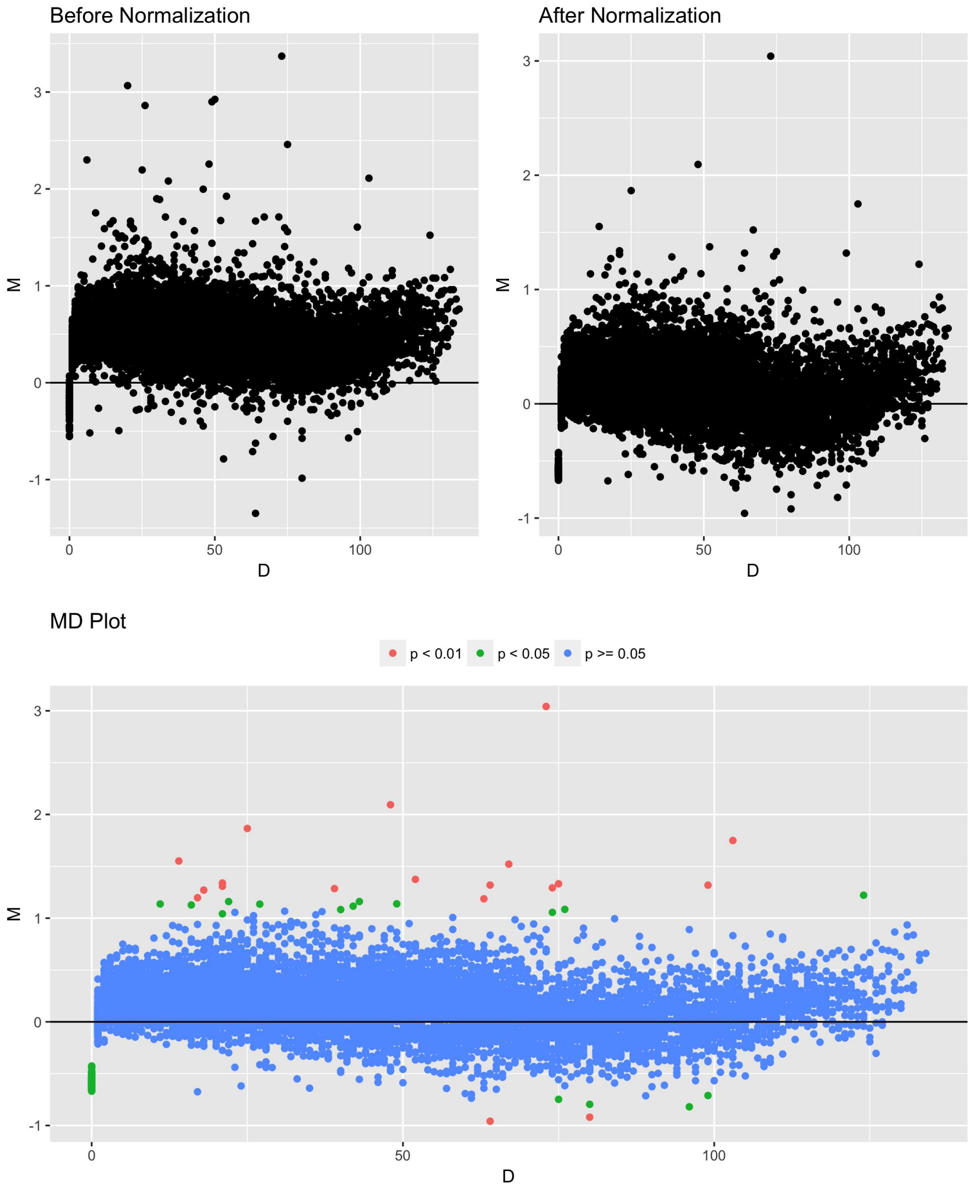


```
[1] "167 differences found between the datasets"
```

KR

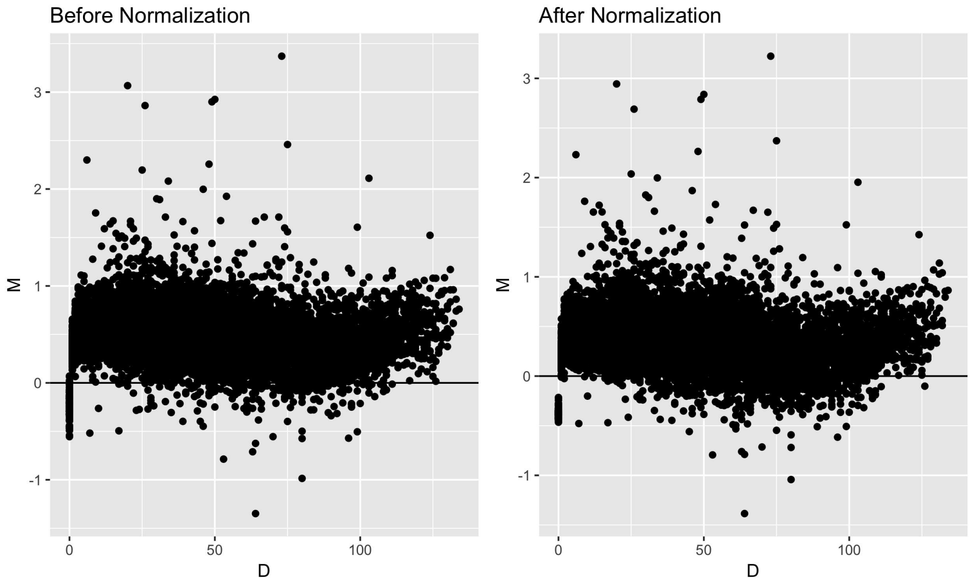

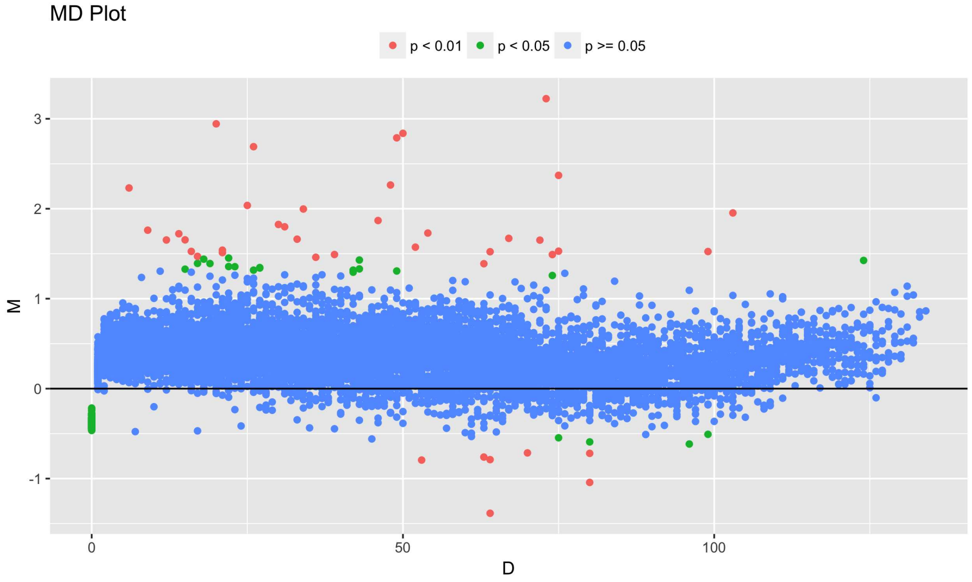


```
[1] "195 differences found between the datasets"
```

SCN

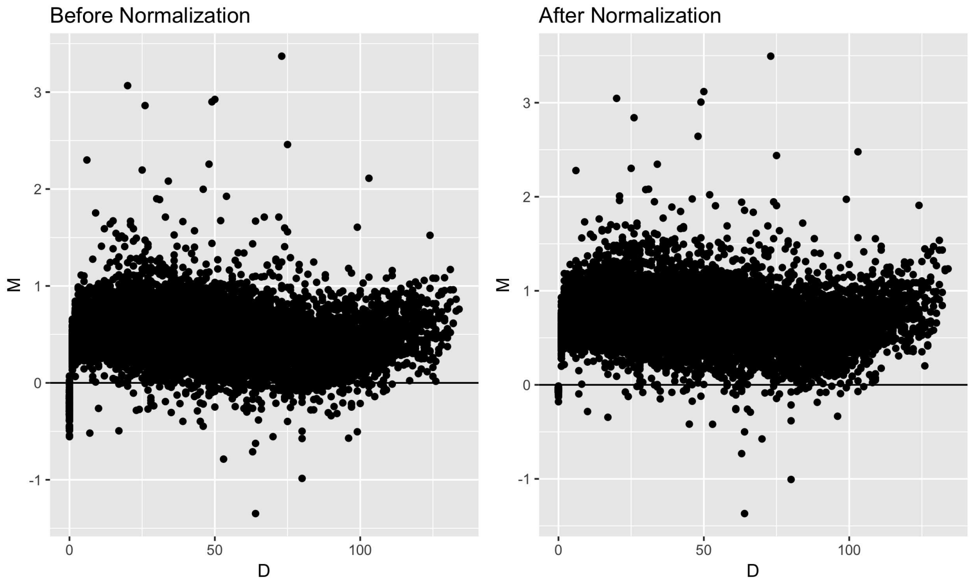

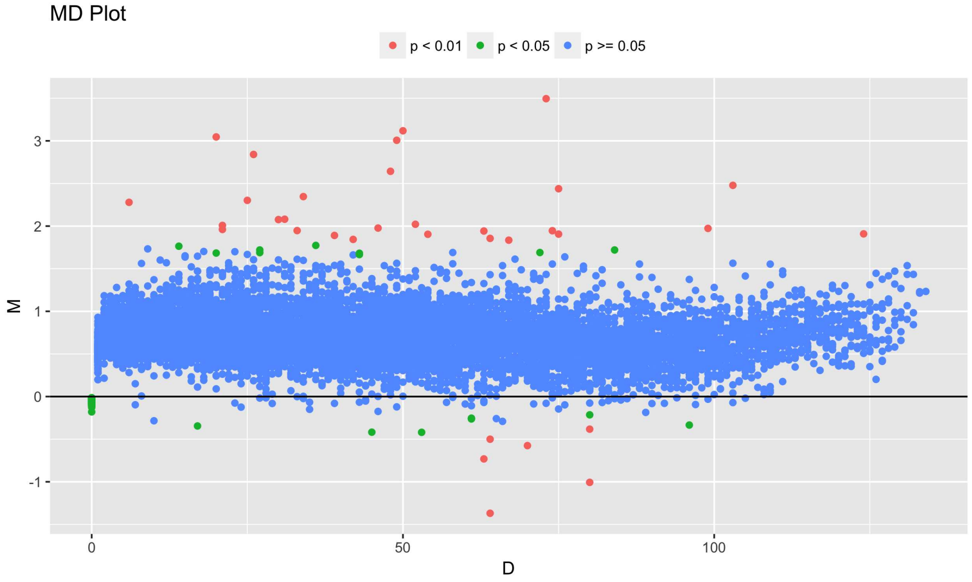


```
[1] "183 differences found between the datasets"
```

Summary

`Loess` is the only method that can successfully remove the bias between the two datasets. The individual normalization techniques fail to remove biases between the datasets though they may be effective at removing bias within a single dataset. `KR` normalization appears to be second to the `loess` normalization in removing global and local biases.

Comparison of `HiCcompare` vs. `ChromoR` in detecting differential chromatin interactions

`ChromoR` includes a function for detecting differences between two Hi-C datasets. Using the data for chromosome 11 from GM12878 as used in the above normalization comparison we add 200 *a priori* known differences to the matrix at a 5 fold change and attempt to detect them using `ChromoR` and `loess`

The MD plot of the `ChromoR` normalized matrices:

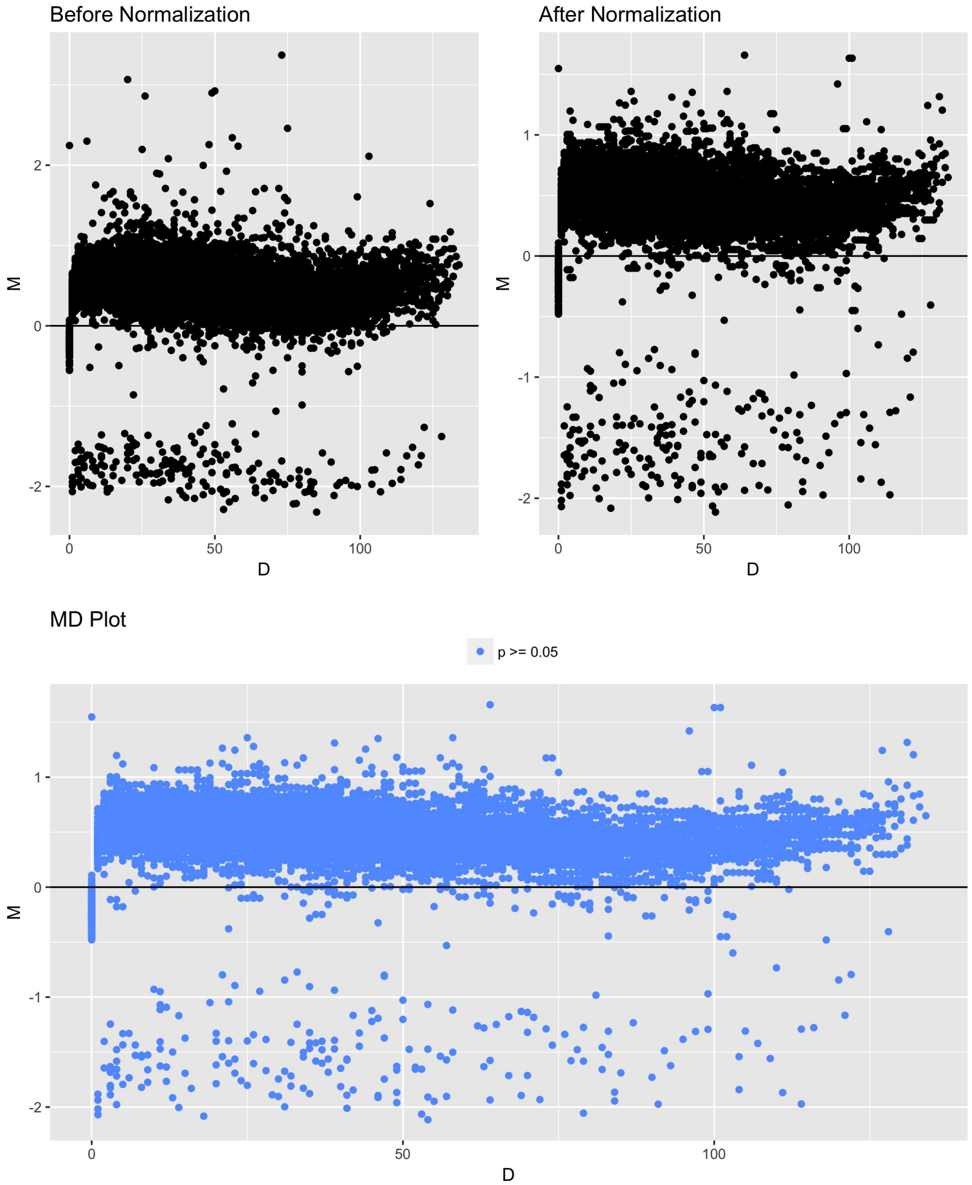

`ChromoR` found 0 differences between the two matrices. Compared to `hic_loess` below:

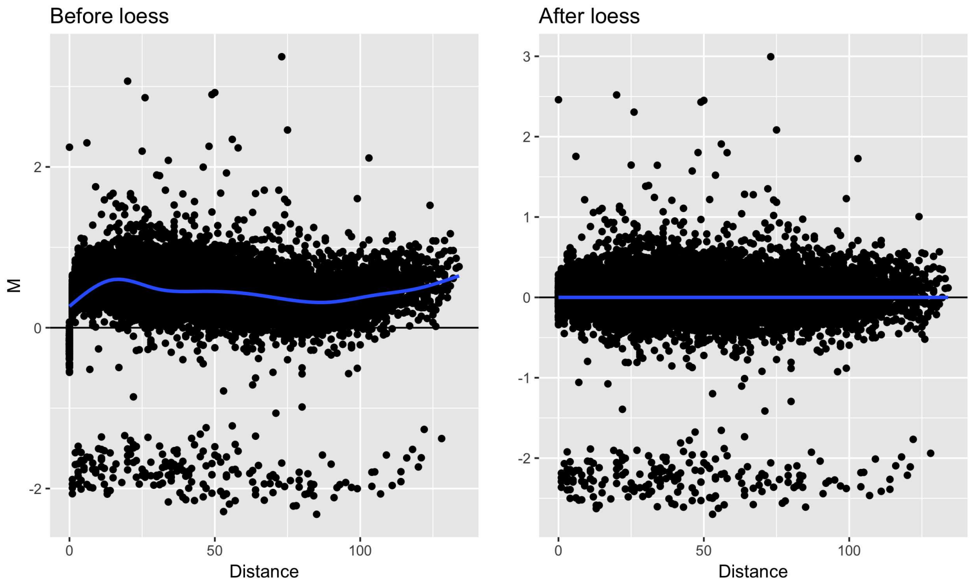

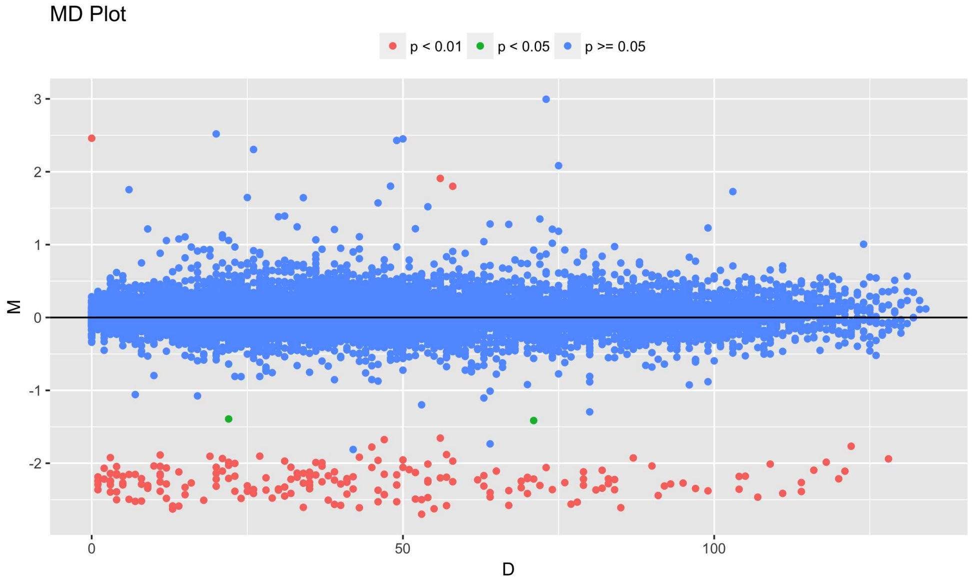

`HiCcompare` found 199 differences between the matrices.

`ChromoR`'s normalization technique fails to remove bias between Hi-C datasets and it's difference detection method also fails to detect any differences when true differences are added at a 5 fold change. `Loess` was capable of normalizing these datasets and detecting the majority of the true differences added to the matrices.

5. Extended evaluation of differential chromatin interaction detection analysis using real Hi-C data

1MB Resolution

Here we evaluate the performance of the differential detection method of `HiCcompare` using replicate Hi-C data, GM12878 cell line, chromosome 1, at 1MB resolution. 200 controlled differences at various fold changes (2, 3, 4) were introduced into the raw data, the matrices were normalized using `HiCcompare` and various individual normalization methods (chromoR, ICE, KR, SCN, MA, see Supplementary Methods). The performance of each normalization method's effect on difference detection was evaluated using the following metrics: "TP" - true positives, "FP" - false positives, "TN" - true negatives, "FN" - false negatives, "TPR" - True Positive Rate, aka recall, or sensitivity $TP/(TP+FN)$, "SPC" - specificity, $TN/(FP+TN)$, "F1" - F_1 score, $2TP/(2TP+FP+FN)$, "AUC" - area under ROC curve, "Accuracy" - $(TP+TN)/(TP+FP+TN+FN)$, "Precision" - $TP/(TP+FP)$, "FPR" - False Positive Rate, $FP/(FP+TN)$, "FNR" - False Negative Rate, $FN/(TP+FN)$, "FOR" - False omission rate, $FN/(FN+TN)$, "NPV" - Negative Predictive Value, $TN/(FN+TN)$, "MCC" - Matthews correlation coefficient, $\frac{TP\times TN-FP\times FN}{\sqrt{(TP+FP)(TP+FN)(TN+FP)(TN+FN)}}$.

Fold change 2

	loess	chromoR	ice	kr	scn	ma
**true positive**	187	31	183	191	186	177
**false positive**	61	170	70	134	109	64
**true negative**	26300	31000	25200	26200	26300	26300
**false negative**	13	169	7	9	14	23
**Total**	26600	31400	25400	26600	26600	26600
**TPR**	0.935	0.155	0.963	0.955	0.93	0.885
**SPC**	0.998	0.995	0.997	0.995	0.996	0.998
**F1**	0.999	0.995	0.998	0.997	0.998	0.998
**AUC**	0.938	0.706	0.965	0.956	0.941	0.896
**AUC 20%**	0.187	0.0674	0.192	0.19	0.186	0.177
**FDR**	0.246	0.846	0.277	0.412	0.369	0.266
**Accuracy**	0.997	0.989	0.997	0.995	0.995	0.997
**Precision**	0.754	0.154	0.723	0.588	0.631	0.734
**FPR**	0.00231	0.00545	0.00277	0.00508	0.00413	0.00243
**FNR**	0.065	0.845	0.0368	0.045	0.07	0.115
**FOR**	0.000494	0.00542	0.000278	0.000343	0.000533	0.000874
**NPV**	1	0.995	1	1	0.999	0.999
**MCC**	0.838	0.149	0.833	0.747	0.764	0.805

Fold change 3

	loess	chromoR	ice	kr	scn	ma
**true positive**	197	79	193	199	193	189
**false positive**	9	142	109	142	163	15
**true negative**	26400	31000	25100	26200	26200	26300
**false negative**	3	121	1	1	7	11
**Total**	26600	31400	25400	26600	26600	26600
**TPR**	0.985	0.395	0.995	0.995	0.965	0.945
**SPC**	1	0.995	0.996	0.995	0.994	0.999
**F1**	1	0.996	0.998	0.997	0.997	1
**AUC**	0.986	0.81	0.996	0.996	0.965	0.95
**AUC 20%**	0.197	0.117	0.199	0.199	0.191	0.189
**FDR**	0.0437	0.643	0.361	0.416	0.458	0.0735
**Accuracy**	1	0.992	0.996	0.995	0.994	0.999
**Precision**	0.956	0.357	0.639	0.584	0.542	0.926
**FPR**	0.000341	0.00455	0.00432	0.00539	0.00618	0.000569
**FNR**	0.015	0.605	0.00515	0.005	0.035	0.055
**FOR**	0.000114	0.00388	3.98e-05	3.81e-05	0.000267	0.000417
**NPV**	1	0.996	1	1	1	1
**MCC**	0.97	0.372	0.796	0.76	0.721	0.935

Fold change 4

	loess	chromoR	ice	kr	scn	ma
**true positive**	199	114	198	200	200	195
**false positive**	1	120	20	65	57	4
**true negative**	26400	31100	25200	26300	26300	26400
**false negative**	1	86	0	0	0	5
**Total**	26600	31400	25400	26600	26600	26600
**TPR**	0.995	0.57	1	1	1	0.975
**SPC**	1	0.996	0.999	0.998	0.998	1
**F1**	1	0.997	1	0.999	0.999	1
**AUC**	0.995	0.884	1	1	1	0.977
**AUC 20%**	0.199	0.148	0.2	0.2	0.2	0.195
**FDR**	0.005	0.513	0.0917	0.245	0.222	0.0201
**Accuracy**	1	0.993	0.999	0.998	0.998	1
**Precision**	0.995	0.487	0.908	0.755	0.778	0.98
**FPR**	3.79e-05	0.00385	0.000793	0.00247	0.00216	0.000152
**FNR**	0.005	0.43	0	0	0	0.025
**FOR**	3.79e-05	0.00276	0	0	0	0.00019
**NPV**	1	0.997	1	1	1	1
**MCC**	0.995	0.524	0.953	0.868	0.881	0.977

100KB Resolution

Here we evaluate the performance of the differential detection method of `HiCcompare` using replicate Hi-C data, GM12878 cell line, chromosome 22, at 100KB resolution. 2000 controlled differences at various fold changes (3, 4) were introduced into the raw data, the matrices were normalized using `HiCcompare` and various individual normalization methods.

Fold change 3

	loess	chromoR	ice	scn	ma
**true positive**	1800	298	879	161	1790
**false positive**	35	180	282	360	42
**true negative**	55600	60300	55200	55300	55600
**false negative**	205	1700	1120	1840	206
**Total**	57700	62500	57400	57700	57700
**TPR**	0.898	0.149	0.44	0.0805	0.897
**SPC**	0.999	0.997	0.995	0.994	0.999
**F1**	0.998	0.985	0.987	0.981	0.998
**AUC**	0.938	0.61	0.969	0.93	0.939
**AUC 20%**	0.186	0.0595	0.188	0.168	0.187
**FDR**	0.0191	0.377	0.243	0.691	0.0229
**Accuracy**	0.996	0.97	0.976	0.962	0.996
**Precision**	0.981	0.623	0.757	0.309	0.977
**FPR**	0.000629	0.00298	0.00509	0.00647	0.000755
**FNR**	0.102	0.851	0.56	0.92	0.103
**FOR**	0.00367	0.0275	0.0198	0.0322	0.00369
**NPV**	0.996	0.973	0.98	0.968	0.996
**MCC**	0.936	0.295	0.567	0.143	0.934

Fold change 4

	loess	chromoR	ice	scn	ma
**true positive**	1880	455	1620	382	1880
**false positive**	10	189	217	236	14
**true negative**	55600	60300	55200	55400	55600
**false negative**	123	1540	382	1620	116
**Total**	57700	62500	57400	57700	57700
**TPR**	0.938	0.228	0.809	0.191	0.942
**SPC**	1	0.997	0.996	0.996	1
**F1**	0.999	0.986	0.995	0.984	0.999
**AUC**	0.944	0.673	0.985	0.95	0.946
**AUC 20%**	0.188	0.0829	0.194	0.176	0.188
**FDR**	0.0053	0.293	0.118	0.382	0.00738
**Accuracy**	0.998	0.972	0.99	0.968	0.998
**Precision**	0.995	0.707	0.882	0.618	0.993
**FPR**	0.00018	0.00313	0.00391	0.00424	0.000252
**FNR**	0.0615	0.772	0.191	0.809	0.058
**FOR**	0.00221	0.025	0.00687	0.0284	0.00208
**NPV**	0.998	0.975	0.993	0.972	0.998
**MCC**	0.965	0.391	0.839	0.332	0.966

50KB Resolution

Here we evaluate the performance of the differential detection method of `HiCcompare` using replicate Hi-C data, GM12878 cell line, chromosome 22, at 50KB resolution. 2000 controlled differences at various fold changes (3, 4) were introduced into the raw data, the matrices were normalized using `HiCcompare` and various individual normalization methods.

Fold change 3

	loess	chromoR	ice	scn	ma
**true positive**	634	529	5	5	0
**false positive**	232	565	1280	1350	572
**true negative**	215000	245000	214000	214000	214000
**false negative**	1370	1470	1990	2000	2000
**Total**	217000	248000	217000	217000	217000
**TPR**	0.317	0.264	0.0025	0.0025	0
**SPC**	0.999	0.998	0.994	0.994	0.997
**F1**	0.996	0.996	0.992	0.992	0.994
**AUC**	0.799	0.739	0.825	0.803	0.828
**AUC 20%**	0.143	0.1	0.131	0.122	0.137
**FDR**	0.268	0.516	0.996	0.996	1
**Accuracy**	0.993	0.992	0.985	0.985	0.988
**Precision**	0.732	0.484	0.00389	0.0037	0
**FPR**	0.00108	0.0023	0.00595	0.00626	0.00266
**FNR**	0.683	0.736	0.997	0.998	1
**FOR**	0.00632	0.00596	0.00925	0.00925	0.00924
**NPV**	0.994	0.994	0.991	0.991	0.991
**MCC**	0.479	0.354	-0.0043	-0.00457	-0.00496

Fold change 4

	loess	chromoR	ice	scn	ma
**true positive**	1530	974	130	63	25
**false positive**	173	557	1230	1370	504
**true negative**	215000	245000	214000	214000	214000
**false negative**	467	1030	1870	1940	1980
**Total**	217000	248000	217000	217000	217000
**TPR**	0.766	0.487	0.065	0.0315	0.0125
**SPC**	0.999	0.998	0.994	0.994	0.998
**F1**	0.999	0.997	0.993	0.992	0.994
**AUC**	0.835	0.832	0.946	0.924	0.982
**AUC 20%**	0.154	0.137	0.174	0.162	0.189
**FDR**	0.101	0.364	0.904	0.956	0.953
**Accuracy**	0.997	0.994	0.986	0.985	0.989
**Precision**	0.899	0.636	0.0957	0.0439	0.0473
**FPR**	0.000805	0.00226	0.00572	0.00638	0.00234
**FNR**	0.234	0.513	0.935	0.968	0.988
**FOR**	0.00217	0.00416	0.00868	0.00899	0.00913
**NPV**	0.998	0.996	0.991	0.991	0.991
**MCC**	0.828	0.554	0.0718	0.0296	0.0197

6. Comparison with `diffHic`

Here we present more in depth results for the comparison of HiCcompare and diffHic. As diffHic only takes input in the form of raw sequencing files we were not able to directly compare the methods on introduced changes to real replicate data. We instead make a comparison with the results presented by Lun (Lun and Smyth 2015) on the analysis of RWPE1 Hi-C data. HiCcompare tended to detect differential interactions with larger fold changes than those detected by diffHic (Supplemental Figure 5.1). Additionally HiCcompare was able to detect differences across the range of genomic differences while diffHic tends to mostly detect differences at short genomic distances (Supplemental Figure 5.2). Supplemental Table 5.1 compares some metrics between HiCcompare and diffHic and Supplemental Table 5.2 displays the results for interactions validated by FISH.

Supplemental Figure 6.1. Comparing the distributions of M and A. The results of the differential analysis of RWPE1 Hi-C data (Rickman et al. 2012) obtained with `diffHiC` were matched with those obtained with `HiCcompare`. The frequency histograms of the log fold changes used in `diffHiC`, corresponding to the M values used in `HiCcompare` (panels A and B) show that the range of the fold changes is greater in `HiCcompare` analysis. The histograms of the log counts per million (log CPM) used in `diffHiC` corresponding to the log A values used in `HiCcompare` show relative similarity of the distributions.

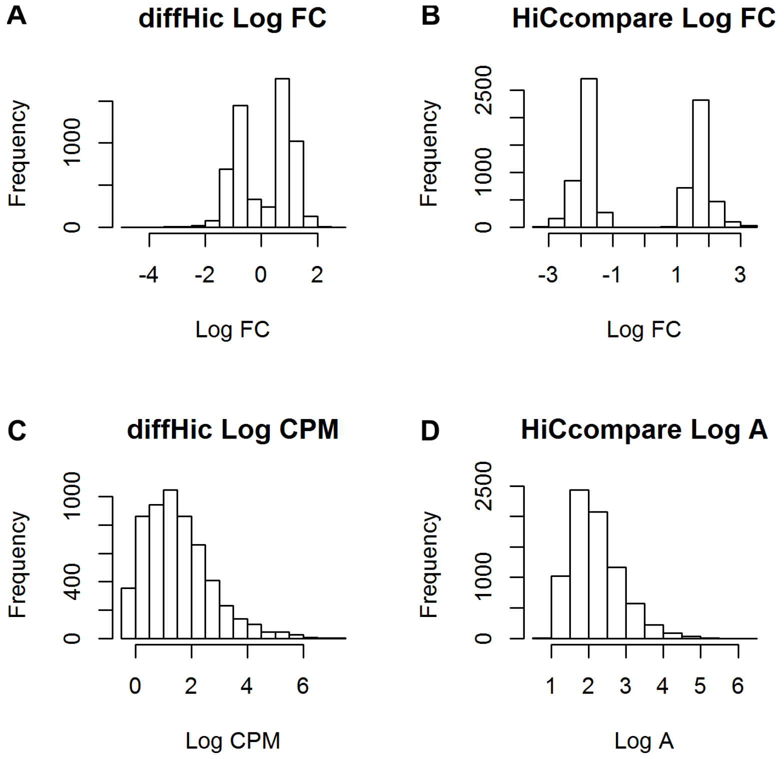

Supplemental Figure 6.2. Comparison of regions detected by HiCcompare and diffHic. Chromosome 1 at 1MB resolution for the comparison of RWPE1 prostate epithelial cells and ERG3 over-expression strains of RWPE1 cells. MD plot showing regions detected as significant by `diffHic` in yellow and regions detected as significant by `HiCcompare` in red.

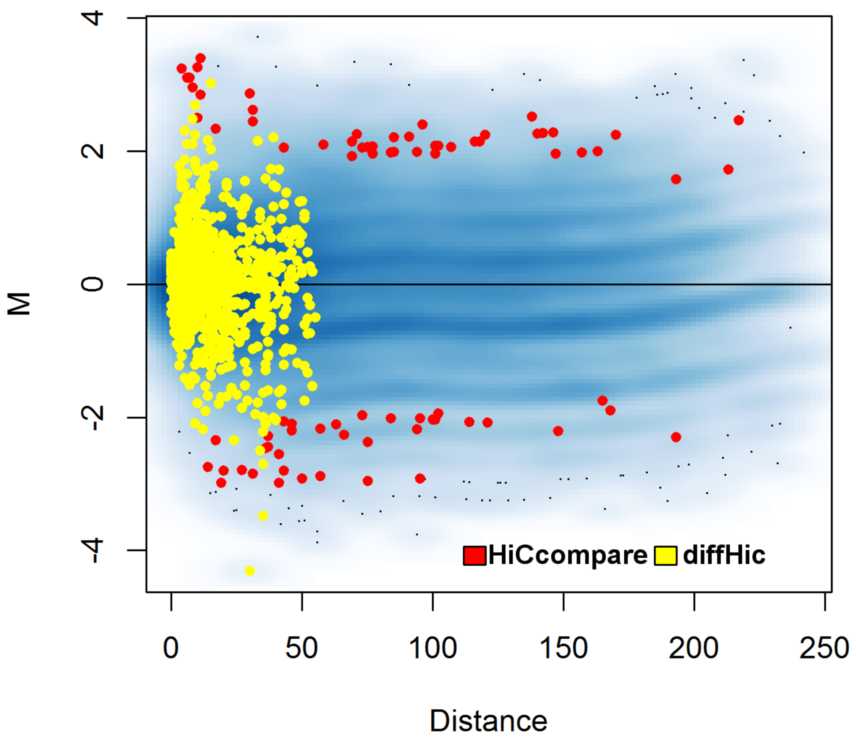

Supplemental Table 6.1. Chromosome-specific comparison of `HiCcompare` and `diffHic` results. "chr" - results are broken down by chromosome; "HiCcompare detected", "diffHic detected" - counts of region pairs detected as significantly differentially interacting by the "HiCcompare" and "diffHiC" pipelines, respectively; "number overlap" - number of region pairs detected as differentially interacting by both methods; "diffHic CNV overlaps", "diffHic blacklist overlaps" - significant regions detected by "diffHiC" method overlapping CNVs and blacklisted regions, respectively; "HiCcomapre mean M+", "HiCcompare mean M-", "diffHic mean M+", "diffHic mean M-" - positive/negative average log M and log fold change in "HiCcompare" and "diffHiC" analyses, respectively.

chr	HiCcompare detected	diffHic detected	number overlap	diffHic CNV overlaps	diffHic blacklist overlap	HiCcompare mean M+	diffHic mean M+	HiCcompare mean M-	diffHic mean M-
chr1	88	344	24	0	115	1.873	0.581	-2.779	-0.626
chr2	65	613	18	0	264	2.499	0.645	-2.838	-0.59
chr3	20	345	4	56	64	1.786	0.586	-2.476	-0.574
chr4	42	306	4	8	96	1.936	0.508	-2.59	-0.541
chr5	59	602	20	0	211	2.284	0.555	-2.675	-0.589
chr6	39	575	23	0	28	2.53	0.781	-2.919	-0.718
chr7	30	324	4	28	122	1.798	0.618	-2.743	-0.653
chr8	28	420	20	81	162	2.175	0.689	-2.646	-0.707
chr9	40	231	7	42	4	1.404	0.528	-2.299	-0.585
chr10	16	302	29	115	124	2.962	1.069	-2.137	-0.596
chr11	38	212	10	0	43	1.889	0.533	-2.762	-0.67
chr12	32	456	7	2	113	2.29	0.745	-2.9	-0.645
chr13	25	174	5	39	71	2.769	0.761	-2.665	-0.587
chr14	34	185	5	0	79	1.963	0.56	-2.477	-0.661
chr15	17	131	5	0	2	2.338	0.755	-2.731	-0.632
chr16	30	58	3	0	2	1.629	0.616	-2.484	-0.651
chr17	16	56	7	0	11	2.038	0.922	-2.83	-0.605
chr18	3	58	0	48	2	1.817	0.489	-1.598	-0.641
chr19	12	37	0	7	17	1.702	0.418	-2.097	-0.672
chr20	21	70	3	1	12	1.715	0.395	-2.293	-0.604
chr21	9	55	0	2	5	1.704	0.412	-1.924	-0.493
chrX	16	180	10	0	2	2.151	0.739	-2.491	-0.661

Four pairs of differentially interacting regions were validated using Fluorescence In Situ Hybridization (FISH) (Rickman et al. 2012) and confirmed in the `diffHiC` analysis (Lun and Smyth 2015). These regions were also detected as differentially interacting in the `HiCcompare` analysis (Table), although they become non-significant after correction for multiple testing due to their relatively small differences. These results suggest that both methods are able to detect biologically relevant chromatin interaction differences, with `HiCcompare` prioritizing large chromatin interaction differences across the full range of distances.

Supplemental Table 6.2. Differential interactions validated by FISH detected by HiCcompare and diffHiC. The regions containing genes listed in the "Interaction" column were previously validated by Fluorescence In Situ Hybridization (FISH) as differentially interacting (Rickman et al. 2012). The "Difference" columns show the differences, measured as $log2(IF_1/IF_2)$, detected by the `HiCcompare` and `diffHiC` pipelines; the "Average" columns show the mean of log interaction frequencies and CPMs; the "p-value/FDR" columns show the raw p-value ("HiCcompare") and FDR-corrected p-values for the corresponding differential interactions. While HiCcompare did not detect any of these interactions after FDR correction, the initial uncorrected p-values were significant for all four interactions. This is likely due to the relative sparsity of the RWPE dataset resulting in a lack of power, especially after multiple-testing correction. It should also be noticed that the average expression values and log2 fold changes for these four interaction are all relatively small as well.

Interaction	HiCcompare difference	HiCcompare average	diffHic difference	diffHic average	HiCcompare p-value	HiCcompare FDR	diffHic FDR
FYN - MOXD1	2.167	8.332	0.733	1.134	0.003	0.112	0.042
HEY2 - MOXD1	2.022	5.297	0.67	2.625	0.006	0.458	0.002
SERPINB9 - MOXD1	-1.712	5.57	-1.27	-0.151	0.025	0.222	0.016
FYN - HEY2	-2.078	8.968	-1.545	0.621	0.006	0.267	0

References

Lun ATL, Smyth GK. 2015. DiffHic: A bioconductor package to detect differential genomic interactions in hi-c data. *BMC Bioinformatics* **16**: 258.

Rickman DS, Soong TD, Moss B, Mosquera JM, Dlabal J, Terry S, MacDonald TY, Tripodi J, Bunting K, Najfeld V, et al. 2012. Oncogene-mediated alterations in chromatin conformation. *Proc Natl Acad Sci U S A* **109**: 9083–8.

7. Comparison with `FIND`

GM12878 vs. K562 comparison

Here we repeat the comparison of GM12878 and K562 presented in the FIND paper (Djekidel et al. 2018) using HiCcompare. The data were obtained from GEO (GSE63525). First we calculated the maximum resolution of each dataset using `Juicer`. Supplemental Table 6.1 lists the maximum resolution of each of the datasets from the analysis. We then plotted the differential interactions detected by HiCcompare and FIND on a single MD plot displayed in Supplemental Fig. 6.1 for chromosome 1 at 1MB resolution. The interactions detected by HiCcompare tended to have larger fold change differences and larger average expression values than those detected by FIND (Supplemental Fig. 6.1 & Supplemental Table 6.3). Additionally FIND detected a much larger number of interactions than HiCcompare at 5KB resolution (Supplemental Table 6.2). However, we believe many of the interactions detected by FIND at 5KB resolution are not very trustworthy due to the sparsity of the data at 5KB resolution and the fact that the maximum resolutions calculated by `Juicer` are much coarser than 5KB. The mean non-zero IF for the GM12878 R1 file was 1.692 and the mean non-zero IF for the GM12878 R2 file was 1.673, further casting doubt on the ability to make any meaningful inferences at this resolution. Additionally we found that FIND takes greater than 72 hours to run on data at resolutions between 100KB and 10KB even in parallel using 16 cores on our department's cluster while HiCcompare is able to complete an analysis in a matter of minutes. For the analysis of the 5KB resolution data FIND took 10 hours and 20 minutes while HiCcompare took 54 minutes. Run times are reported in wall time and were both measured using parallel processing on 16 cores of our department's cluster.

Supplemental Table 7.1. Maximum resolution for Hi-C data. Maximum resolution for each dataset used in the GM12878 vs K562 comparison as calculated by the `calculate_map_resolution.sh` script from `Juicer`.

Data Name	GEO Sample	Maximum Resolution
K562 R1	GSM15551620	38.6 KB
K562 R2	GSM1551623	38.7 KB
GM12878 R1	GSM1551574	8.7 KB
GM12878 R2	GSM1551575	8.4 KB

Supplemental Figure 7.1. Comparison of HiCcompare detected regions and FIND detected regions for GM12878 vs K562. Interactions detected by HiCcompare are shown in red, interactions detected by FIND are shown in yellow. Data shown after HiCcompare's loess normalization. VC Square root normalized data was used for the FIND analysis and the raw data was input into the HiCcompare analysis. Significant interactions detected by FIND tend to have small fold changes while differences detected by HiCcompare have much larger fold changes. Chr 1 of GM12878 vs. K562 at 1MB resolution.

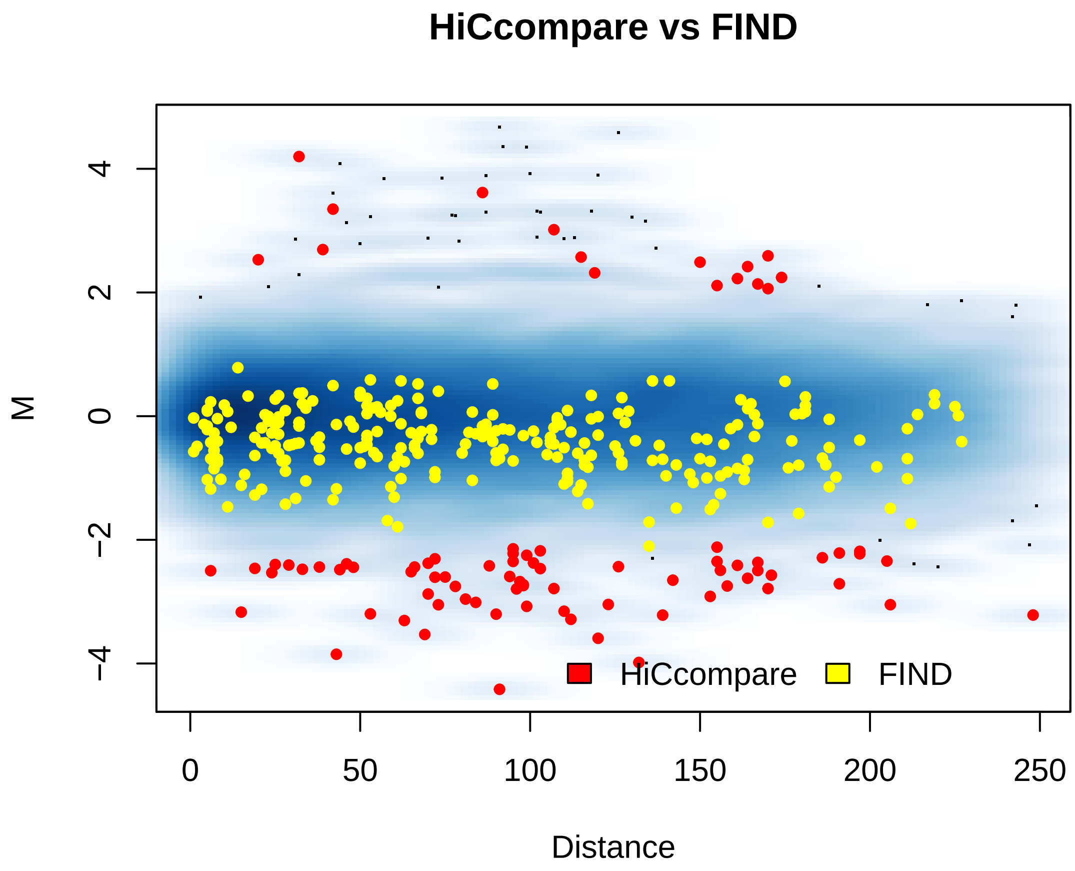

Supplemental Table 7.2. Number of regions detected as significant by chromosome and resolution for the HiCcompare analysis of GM12878 vs K562. The number of interactions detected start dropping off after 50KB due to the increasing sparsity of the data.

chr	1MB	100KB	50KB	10KB	5KB
chr1	86	138	270	111	7
chr2	111	127	123	18	0
chr3	42	54	85	12	0
chr4	139	135	143	20	1
chr5	52	99	116	36	0
chr6	86	191	206	67	6
chr7	93	101	116	53	0
chr8	67	133	110	21	1
chr9	56	83	93	74	7
chr10	17	64	53	10	1
chr11	28	65	88	18	2
chr12	46	89	45	18	0
chr13	40	457	924	3	3
chr14	35	47	42	13	0
chr15	26	74	95	12	0
chr16	21	67	85	25	0
chr17	19	29	60	15	3
chr18	15	66	53	11	0
chr19	17	22	47	16	0
chr20	10	33	32	8	2
chr21	11	41	89	13	0
chr22	14	223	111	0	0
chrX	54	27	30	3	0

Supplemental Table 7.3. Summary of M and A values for interaction detected by HiCcompare and FIND. The Mean M values (log2 fold change) were split into two groups, those above 0 and those below 0. HiCcompare detected differences with larger fold changes than those detected by FIND. Additionally the average expression for the interactions detected by HiCcompare were larger than those detected by FIND.

Measure	HiCcompare	FIND
Mean M < 0	-2.873840	-0.8594336
Mean M >= 0	2.563457	0.3425217
Mean A	778.516303	682.4690639

Comparison on data with a priori known differences

We performed a comparison of HiCcompare and FIND by adding in a priori known differences to GM12878 replicate data as described in the methods. This was performed at fold changes of 2, 3, and 5 on 1MB data for chr 18. 200 true differences were introduced at the specified fold changes between the two replicate datasets. Each dataset was then run through HiCcompare and FIND and standard performance classifiers were assessed. These results are listed in tables 6.4 - 6.6. Note that the total numbers differ between the HiCcompare and FIND columns due to the fact the HiCcompare ignores cells of the matrix with 0's while they are included in FIND. Classifiers are denoted by "TP" - true positives, "FP" - false positives, "TN" - true negatives, "FN" - false negatives, "TPR" - True Positive Rate, aka recall, or sensitivity $TP/(TP + FN)$, "SPC" - specificity, $TN/(FP + TN)$, "Accuracy" - $(TP + TN)/(TP + FP + TN + FN)$, "Precision" - $TP/(TP + FP)$, "FPR" -

False Positive Rate, $FP/(FP+TN)$, "FNR" - False Negative Rate, $FN/(TP+FN)$, "FOR" - False omission rate, $FN/(FN+TN)$, "NPV" - Negative Predictive Value, $TN/(FN+TN)$, "MCC" - Matthews correlation coefficient, $\frac{TP \times TN - FP \times FN}{\sqrt{(TP+FP)(TP+FN)(TN+FP)(TN+FN)}}$.

Supplemental Table 7.4. HiCcompare vs FIND on 2 fold change introduced differences.

	HiCcompare	FIND
true positive	183.000	14.000
false positive	0.000	156.000
true negative	2799.000	5885.000
false negative	17.000	186.000
Total	2999.000	6241.000
TPR	0.915	0.070
SPC	1.000	0.974
FDR	0.000	0.918
Accuracy	0.994	0.945
Precision	1.000	0.082
FPR	0.000	0.026
FNR	0.085	0.930
FOR	0.006	0.031
NPV	0.994	0.969
MCC	0.954	0.048

Supplemental Table 7.5. HiCcompare vs FIND on 3 fold change introduced differences.

	HiCcompare	FIND
true positive	191.000	4.000
false positive	0.000	21.000
true negative	2799.000	6020.000
false negative	9.000	196.000
Total	2999.000	6241.000
TPR	0.955	0.020
SPC	1.000	0.997
FDR	0.000	0.840
Accuracy	0.997	0.965
Precision	1.000	0.160
FPR	0.000	0.003
FNR	0.045	0.980
FOR	0.003	0.032
NPV	0.997	0.968
MCC	0.976	0.046

Supplemental Table 7.6. HiCcompare vs FIND on 5 fold change introduced differences.

	HiCcompare	FIND
true positive	191.000	4.000
false positive	0.000	25.000
true negative	2799.000	6016.000
false negative	9.000	196.000
Total	2999.000	6241.000
TPR	0.955	0.020
SPC	1.000	0.996
FDR	0.000	0.862
Accuracy	0.997	0.965
Precision	1.000	0.138
FPR	0.000	0.004
FNR	0.045	0.980
FOR	0.003	0.032
NPV	0.997	0.968
MCC	0.976	0.041

References

Djekidel MN, Chen Y, Zhang MQ. 2018. FIND: DifFerential chromatin interactions detection using a spatial poisson process. *Genome Res.*
